# Fatal risk factors and the efficacy of glucocorticoid therapy in severe fever with thrombocytopenia syndrome: a multicenter retrospective cohort study

**DOI:** 10.3389/fcimb.2025.1531880

**Published:** 2025-05-08

**Authors:** Fang Zhong, Shiyu Zhang, Chengxi Zheng, Dilihumaer Zhayier, Shuting Liu, Qinyu You, Hongming Huang, Bin Zhu, Jin Tian, Zhiliang Hu, Xin Zheng, Baoju Wang, Zhihang Peng

**Affiliations:** ^1^ School of Public Health, Nanjing Medical University, Nanjing, China; ^2^ Department of Infectious Disease, Union Hospital, Tongji Medical College, Huazhong University of Science and Technology, Wuhan, China; ^3^ Department of Infectious Disease, the Second Hospital of Nanjing, School of Public Health, Nanjing Medical University, Nanjing, China; ^4^ Nanjing Hospital, Nanjing University of Chinese Medicine, Nanjing, China; ^5^ Center for Global Health, School of Public Health, Nanjing Medical University, Nanjing, China; ^6^ National Key Laboratory of Intelligent Tracking and Forecasting for Infectious Diseases, Chinese Center for Disease Control and Prevention, Beijing, China; ^7^ Chinese Center for Disease Control and Prevention, Beijing, China

**Keywords:** nomogram, prediction model, severe fever with thrombocytopenia syndrome, case fatality rate, glucocorticoids

## Abstract

**Background:**

Severe fever with thrombocytopenia syndrome (SFTS) is an emerging tick-borne infectious disease characterized by rapid progression and high mortality. Glucocorticoids (GCs) can be used as anti-inflammatory agents for SFTS, but no standardized protocols have been proposed.

**Methods:**

A total of 901 patients with SFTS diagnosed at two hospitals between July 2017 and October 2023 were included in this retrospective cohort study. Univariate and multivariate logistic regression were performed along with LASSO regression to identify independent risk factors of fatal outcomes and further develop mortality prediction model. A nomogram was used to visualize the predictive model. ROC curves, calibration curves, and DCA curves were conducted to assess model accuracy and clinical applicability. The efficacy of GC was assessed using survival analyses, and further subgroup analyses of the effects of different GC regimens on fatal outcomes and hospital-acquired infections (HAI) were performed. Propensity score matching (PSM) analyses were conducted to control confounding factors.

**Results:**

Older age (age > 69 years), consciousness disturbance, decreased monocyte counts, prolonged activated partial thromboplastin time (APTT), and high viral load were identified as strong predictors of fatal outcomes in patients with SFTS. Patients were classified into mild and severe groups according to risk scores calculated by the nomogram (cut-off value = 121.43). Survival analyses showed that GCs treatment may reduce the mortality in severe patients (p = 0.004). Further subgroup analyses indicated that relatively high doses and early treatment with GCs may increase mortality in SFTS patients [OR = 2.292 (1.071, 5.066); OR = 3.693 (1.710, 8.345) respectively]. GCs treatment was associated with an elevated risk of HAI in patients both with mild and severe SFTS (p = 0.024; p = 0.015, respectively). Initiation of GCs therapy at a low level of aspartate aminotransferase (AST < 189.75 U/L) reduced the mortality before and after PSM (p<0.001; p = 0.004, respectively).

**Conclusions:**

A new nomogram based on five independent risk factors effectively predicts the prognosis of SFTS. Severe patients and those with low AST levels might benefit from GCs therapy while early and relatively high doses of GCs therapy should be used with caution.

## 1 Introduction

Severe fever with thrombocytopenia syndrome (SFTS) is an emerging tick-borne infectious disease caused by *Dabie bandavirus* (formerly known as SFTS virus [SFTSV]) (Kim and Park, 2023). The first case of SFTS in China was confirmed in 2009 (Yu et al., 2011), followed by a total of 7,721 laboratory-confirmed SFTS cases during 2010-2018, with an overall case fatality rate (CFR) of 10.5%. Despite this high CFR, no specific antiviral therapy has been established.

Previous studies and meta-analyses have shown that among clinical symptoms and laboratory markers of SFTS, age, emesis, neurologic symptoms, disseminated Intravascular Coagulation (DIC), multiorgan dysfunction, and shock were significantly associated with death, whereas its risk also increases significantly in those with abnormalities in viral load, prothrombin time (TT), activated partial thromboplastin time (APTT), percentage of mononuclear cells, percentage of lymphocytes, and coagulation and liver function (Wang et al., 2022a; Wang et al., 2022b; Wang et al., 2024). Thus, early risk stratification is critical to improve patients’ prognosis. Models based on a joint of indices have been developed to predict mortality in patients with SFTS, but most of them are single-centered and size-limited, which in turn restricts their clinical applications (Jia et al., 2017; Katsurada et al., 2017; Wei X. et al., 2022). In a recent multicenter study, 377 patients with SFTS were divided into double-positive, single-positive, and double-negative groups based on their neurological symptoms; a model incorporating age, gastrointestinal bleeding, and SFTS viral load (AUC=0.859) was generated for assessing the effectiveness of ribavirin, antibiotics, and gamma globulin (Xia et al., 2023). Nevertheless, the sample size of this study was still limited, and the efficacy of GCs, a common anti-inflammatory agent in the treatment of SFTS, was not evaluated.

The poor prognosis of SFTS may be attributed to high viral load, cytokine storm, and immune dysfunction (Chinese Society of Infectious Diseases and Chinese Medical Association, 2022). Consequently, therapeutic strategies can only be efficacious when bearing antiviral, anti-inflammatory, and immunostimulatory properties. Systemic GCs combat inflammation by inhibiting pro-inflammatory genes and inflammatory cytokines, making them potentially effective against diseases correlated with cytokine release (FakhriRavari et al., 2021). However, GCs-induced immunosuppression may also delay viral clearance and increase the risk of secondary infection (Shang et al., 2020). Guideline issued by the National Health and Wellness Commission of China recommend systemic application of glucocorticoids as adjunctive therapy for patients with severe SFTS, but it not contain criteria for identifying patients suitable for SFTS treatment and specific treatment regimens with optimal clinical efficacy (Chen et al., 2022). This may lead to inappropriate use of GCs in the treatment of SFTS, resulting in adverse outcomes for patients.

Therefore, this study aimed to establish a new model to early identify patients at high risk of death and predict the effect of GCs therapy.

## 2 Materials and methods

### 2.1 Study design and patient enrollment

This retrospective cohort study included patients with SFTS admitted at the Union Hospital of Tongji Medical College, Huazhong University of Science and Technology (Wuhan, China) from May 2020 to October 2023, and the Second Hospital of Nanjing (Nanjing, China) from January 2017 to June 2023. SFTS was laboratory-confirmed according to criteria released by the National Health Commission of China (2010 version) (Ministry of Health, PRC, 2011): (1) an exposure history of previous field activities in SFTS-endemic areas or tick bites within two weeks before febrile symptom onset, acute fever with thrombocytopenia and/or leukopenia); (2) detection of SFTSV RNA by reverse-transcriptase PCR (RT-PCR). Exclusion criteria included: (1) age under 18 years; (2) length of hospitalization less than 48 hours; (3) missing important laboratory results; (4) presence of autoimmune disease, acquired immunodeficiency syndrome (AIDS), and malignant tumor (see **Supplementary Figure 1**).

This study was approved by the Ethics Committee of Union Hospital of Tongji Medical College, Huazhong University of Science and Technology (Ethics No. (2021) 1047-01) and the Ethics Committee of Nanjing Medical University, China (Ethics No. (2020) 211) according to the Declaration of Helsinki. This study was restricted to secondary data analysis, so the requirement for informed consent from participants was waived.

### 2.2 Data collection and study definitions

This retrospective analysis was based on patients’ demographic and epidemiological data, clinical manifestations, initial laboratory results following admission, and treatment-related information. SFTS onset was marked as the first occurrence of fever or thrombocytopenia. Endpoint was set as discharge or a fatal outcome, the primary outcome as 28-day mortality, and the secondary outcome as hospital-acquired infection (HAI). HAI was determined if the infection was neither present nor incubating at admission, but occurred within ≥48 h after admission for SFTS (Ge et al., 2022). Systemic GCs therapy was defined as at least one dose of GCs administered via intravenous routes. Considering that dexamethasone is the most commonly prescribed, other types of GCs were converted to dexamethasone equivalents. According to a previous study, the doses of methylprednisolone, dexamethasone, and hydrocortisone were comparable, with a conversion ratio of 1:5.3 for dexamethasone to methylprednisolone and 5:1 for hydrocortisone to methylprednisolone (Wang et al., 2023). The timing (classified as early (≤ 6 days) and late (>6 days from onset to GCs use)), duration (classified as long-term (> 3 days) and short-term (≤ 3 days)), and dose of GCS treatment (classified as high (> 5 mg/day) and low (≤ 5 mg/day dexamethasone or its equivalents)) were defined based on median number of patients in the multicenter cohort.

### 2.3 Statistical analysis

As many eligible cases as possible were included due to the exploratory nature of the study. Variables with more than 20% missing data were removed and other missing cases were resolved by multiple interpolation. Data normality was assessed using the Kolmogorov-Smirnov test. Continuous variables were expressed as medians (interquartile range [IQR]), and categorical variables were expressed as frequencies (percentages). For continuous data, differences between groups were measured using the independent samples t-test or Mann-Whitney U-test, as appropriate. Categorical data were analyzed using the Pearson chi-square test or Fisher’s exact test.

This study involved a large number of variables, and to screen for potential predictors, least absolute shrinkage and selection operator (LASSO) regression and univariate logistic regression were used. The screened variables were subsequently incorporated into multivariate logistic regression using stepwise regression to identify independent predictors of death in patients with SFTS. A prediction model was developed with all patients’ data (avoiding data segmentation), and internal validation of model discrimination and calibration was performed using bootstrapping with 1000 resamples (Riley et al., 2020). Receiver operating characteristics (ROC) curves and calibration curves were used to determine the accuracy and consistency of the model. The overall benefit of the model was demonstrated by Decision Curve Analysis (DCA). Kaplan-Meier curves and log-rank tests were used to compare the survival rate and survival time. To reduce the effects of selection bias and potential confounders, we also performed propensity score matching (PSM) in a 1:1 ratio to divide the patients into two risk groups based on the logit of the propensity score (PS) using nearest-neighbor matching with a caliper width of 0.1. After PSM, subgroup analyses were performed based on patient characteristics and treatment regimens. Data processing and statistical analysis were performed in R software (version 4.3.2). All tests were two-tailed, with p-values less than 0.05 considered significant.

## 3 Results

### 3.1 Demographic and clinical data of SFTS patients

Of the 901 patients with SFTS, 105 patients (50 males and 55 females) died, with an overall mortality rate of 11.65%. The median age of the patients was 64.00 (56.00-70.00) years, and the median time from symptom onset to hospital admission was 6.0 (4.0-7.0) days. Fever, diarrhea, vomiting, muscle pain, and cough were the most common symptoms. Basic clinical indicators, such as gender, age, white blood cell (WBC) count, platelets, and log-transformed viral load (lg viral load), were compared between the survival and fatal groups. Compared with the survival group, the fatal group had an older age, a shorter time from onset to admission, and higher incidences of consciousness disturbance, hypertension, myalgia, and hemorrhagic tendencies (see **Supplementary Table 1**).

### 3.2 Risk factors for fatal outcomes

A total of 53 variables were measured at hospital admission (see **Supplementary Figure 3**). After LASSO regression (see **Supplementary Figure 2**), 12 variables remained significant between survival and fatal groups, including older age, consciousness disturbance, WBC, decreased monocyte counts, SCr, BUN, LDH, INR, prolonged APTT, PCT, bleeding, and lg viral load. Subsequently, the univariate regression analysis showed that older age (>69 years), time from onset to admission, consciousness disturbance, diarrhea, muscle pain, white blood cell (WBC) count, lymphocyte count, decreased monocyte count (<0.12×10^9^/L), platelet count (PLT), aspartate aminotransferase (AST), GLOB, serum creatinine (SCr), blood urea nitrogen (BUN), lactate dehydrogenase (LDH), creatine phosphokinase (CK), calcium (Ca), D-dimer, international normalized ratio (INR), prolonged APTT (>53 s), fibrinogen (FIB), bleeding and lg viral load were screened out as independent predictors (all p < 0.05) (**Table 1**).

**Table 1:** Univariate and multivariate logistic regression analyses of risk factors for fatal outcomes in patients with SFTS.

Variables	AUC	Univariate logistic regression		Multivariate logistic regression	
		OR (95%CI)	p-Value	aOR (95%CI)	p-Value
Old age	0.640	3.314 (2.191, 5.034)	<0.001	2.857 (1.694, 4.820)	<0.001
Time from onset to admission	0.584	0.895 (0.817, 0.973)	<0.05		
Consciousness disturbance	0.805	20.303 (12.697, 33.266)	<0.001	12.994 (7.625, 22.142)	<0.001
Diarrhea	0.553	1.532 (1.018, 2.307)	<0.05		
Muscle pain	0.571	0.418 (0.228, 0.716)	<0.05		
WBC	0.599	0.894 (0.818, 0.966)	<0.05		
Lymphocytes	0.677	0.355 (0.202, 0.583)	<0.001		
Decreased monocyte counts	0.664	3.916 (2.545, 6.150)	<0.001	3.486 (2.022, 6.008)	<0.001
PLT	0.659	0.976 (0.966, 0.986)	<0.001		
AST	0.688	1.001 (1.001, 1.001)	<0.001		
GLOB	0.582	1.043 (1.011, 1.075)	<0.05		
SCr	0.693	1.01 (1.006, 1.014)	<0.001		
BUN	0.692	1.091 (1.045, 1.139)	<0.001		
LDH	0.672	1.001 (1.001, 1.001)	<0.001		
CK	0.650	1.000 (1.000, 1.000)	<0.05		
Ca	0.653	0.059 (0.017, 0.195)	<0.001		
D-dimer	0.646	1.040 (1.021, 1.063)	<0.001		
INR	0.704	33.454 (8.652, 142.429)	<0.001		
Prolonged APTT	0.631	3.089 (2.042, 4.685)	<0.001	2.547 (1.503, 4.316)	<0.001
FIB	0.689	0.349 (0.234, 0.507)	<0.001		
Bleeding	0.594	3.504 (2.157, 5.603)	<0.001		
Lg viral load	0.745	1.597 (1.433, 1.791)	<0.001	1.393 (1.218, 1.594)	<0.001

AUC, area under the curve; OR, odds ratio; aOR, adjusted odds ratio; CI, confidence interval; PLT, platelet count; AST, aspartate aminotransferase; GLOB, globulin; SCr, serum creatinine; BUN, blood urea nitrogen; LDH, lactate dehydrogenase; CK, creatine phosphokinase; Ca, calcium; INR, international normalized ratio; APTT, activated partial thromboplastin time; FIB, fibrinogen; Lg viral load, log-transformed viral load.

### 3.3 Predictive value of the nomogram

Based on the results of the multivariable analysis, five variables were incorporated into the nomogram, including older age, consciousness disturbance, decreased monocyte counts, prolonged APTT, and lg viral load. In this nomogram, the risk score was calculated using the regression equation formula:


Nomogram score=1.050∗old age+2.564∗consiousness disturbance+1.249∗decreased monocyte counts+0.935∗prolonged APTT+ 0.331∗lg(viral load)

**Figure 1:**
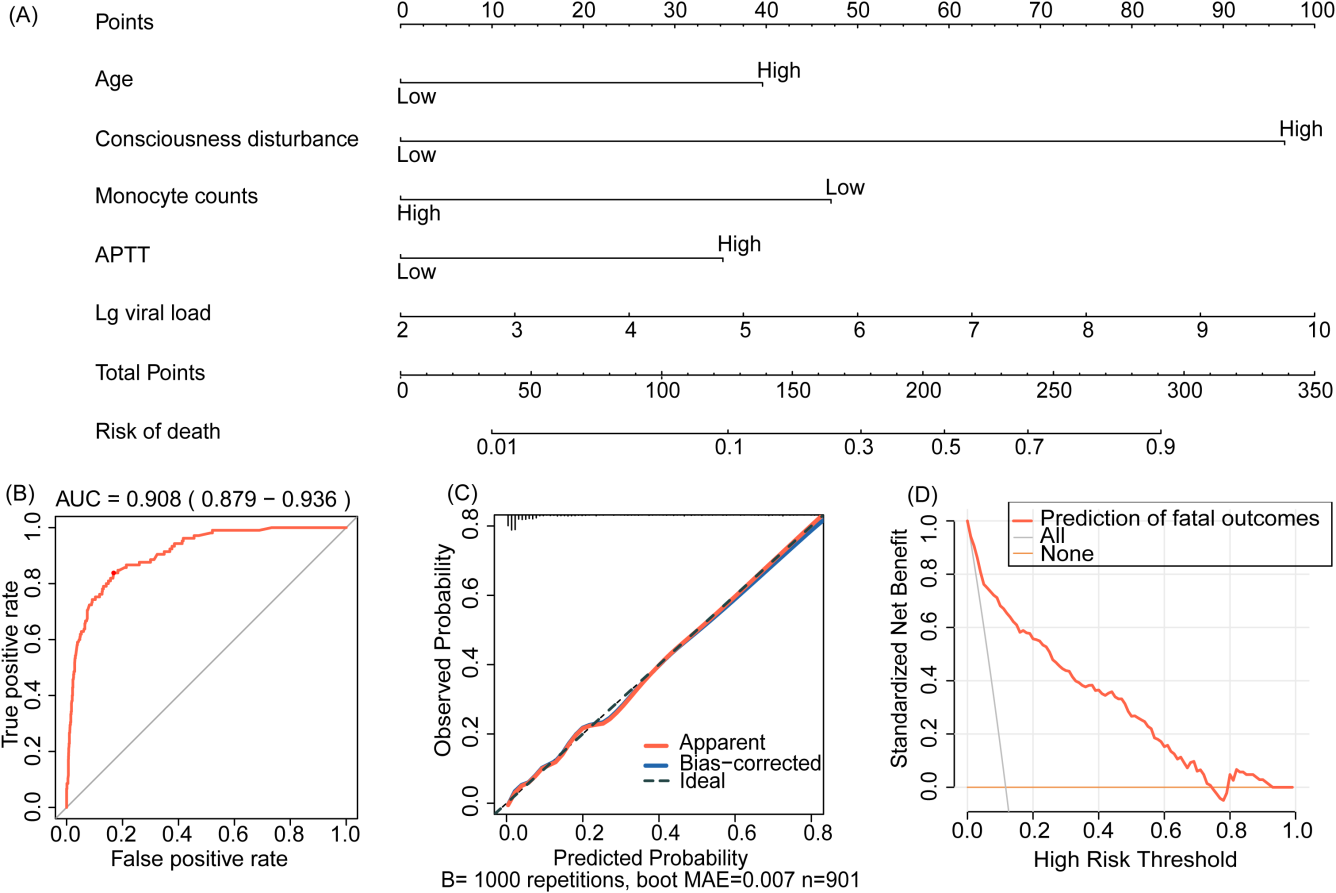
**(A)** The nomogram for predicting fatal outcomes in patients with SFTS. **(B)** Receiver operating characteristics (ROC) curve, **(C)** calibration curves, and **(D)** decision curve analysis (DCA) for the prediction model. Lg viral load, log-transformed viral load; APTT, activated partial thromboplastin time.

After logistic regression analysis (**Table 1**), the five significant and independent variables were incorporated into the nomogram (R^2^ = 0.483, C-index = 0.908) (**Figure 1A**). Its predictive accuracy was evaluated with ROC curves and calibration plots. The ROC curve produced an AUC of 0.908 (95% CI: 0.879-0.936) (**Figure 1B**). The calibration curve was close to the standard diagonal (**Figure 1C**). The DCA showed excellent net benefits of the nomogram (**Figure 1D**), indicating its high concordance and reliability.

### 3.4 Effect of GCs on mild and severe SFTS patients

**Figure 2:**
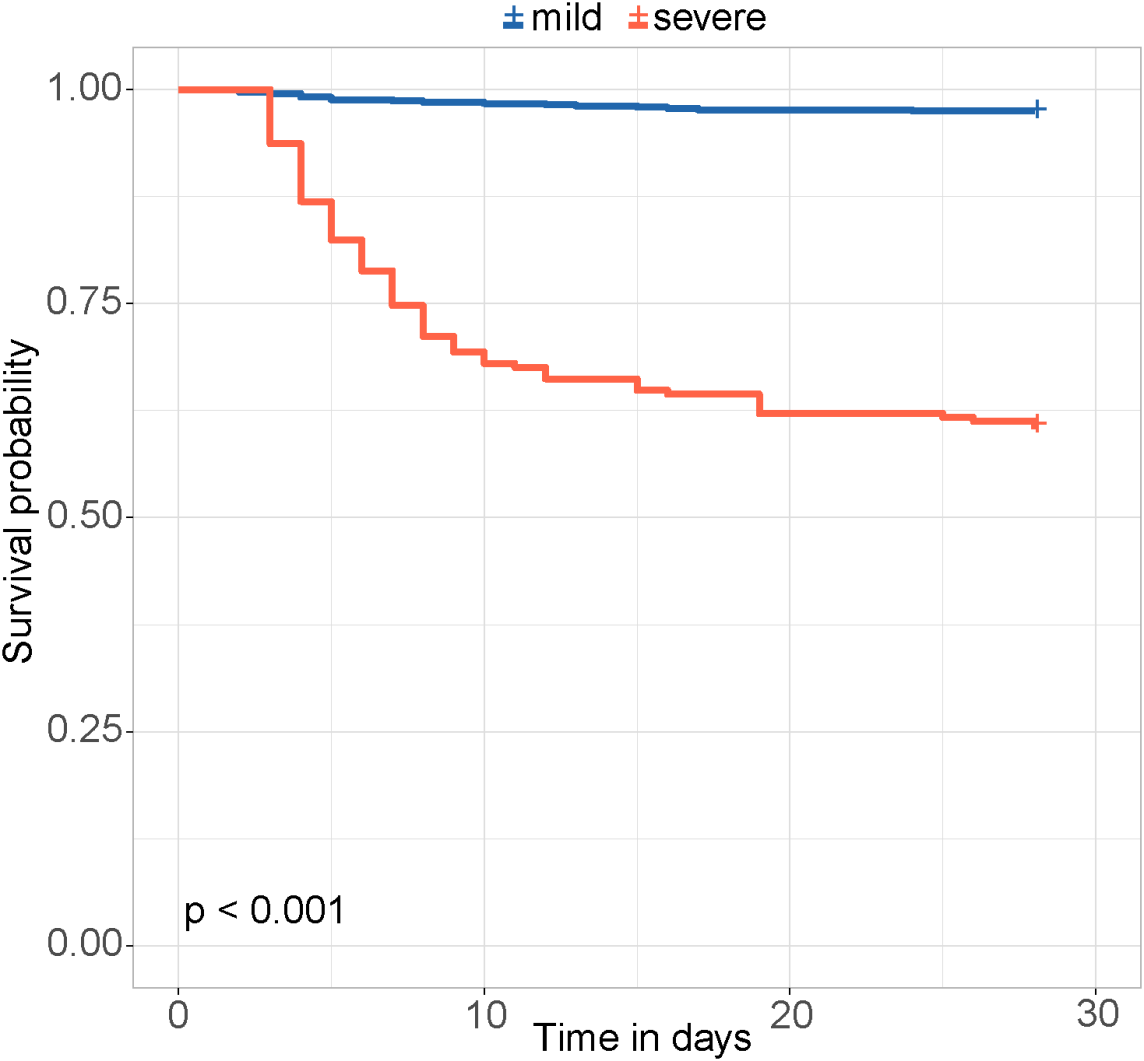
Kaplan-Meier survival curves in mild and severe groups.

**Figure 3:**
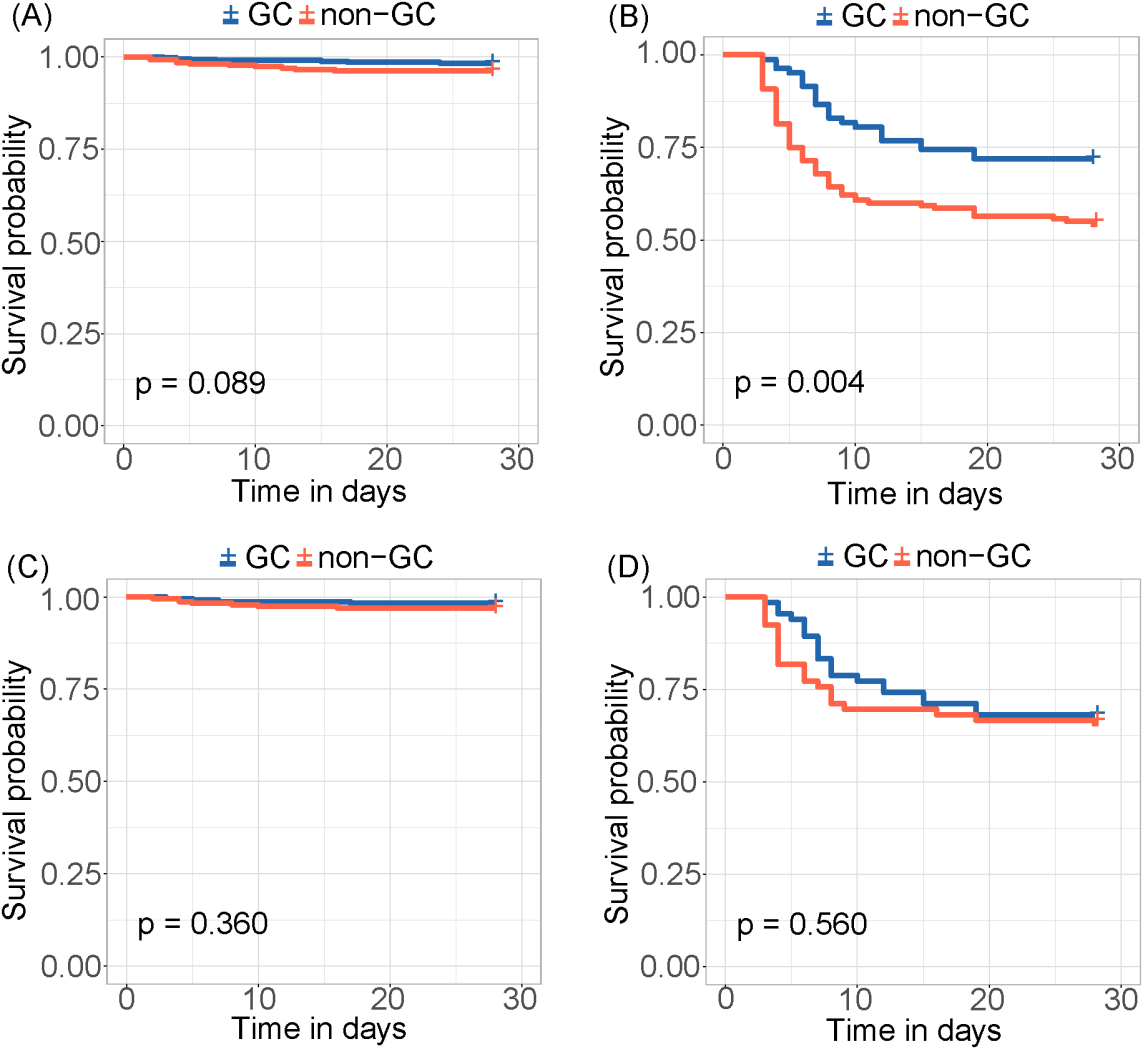
Kaplan-Meier survival curves stratified by GCs treatment for **(A)** mild patients and **(B)** severe patients. Kaplan-Meier survival curves stratified by GCs treatment for **(C)** mild patients and **(D)** severe patients after PSM.

The risk score was calculated for each patient, with a higher score indicating a higher risk of a fatal outcome. According to a cut-off value of 121.434, all patients were categorized into mild and severe groups. The Kaplan-Meier curve showed a significant difference in the survival rate between the mild and severe groups (p < 0.001) (**Figure 2**). In the comparative analysis of patients treated with and without GCs, the mortality and co-infection rates were significantly different between the two groups (see **Supplementary Table 2**). The impacts of GCs were further analyzed in patients with mild and severe SFTS. Before and after PSM, the total CFRs were 2.5% (17/679) and 2.3% (11/474) in patients with mild SFTS, and 39.6% (88/222) and 33.3% (44/132) in patients with severe SFTS, respectively. After PSM, 474 patients with mild SFTS and 132 patients with severe SFTS were analyzed, and the baseline characteristics in the GC group were comparable to those in the non-GC group (see **Supplementary Table 3**). Survival analyses showed no significant difference in CFR between the GC and non-GC groups before and after PSM in mild patients (p = 0.089, p = 0.360, **Figures 3A, C**). In contrast, in patients with severe SFTS, CFR was significantly higher in the non-GC group than in the GC group before PSM (p = 0.004, **Figure 3B**), however, significance disappeared after PSM (p = 0.560, **Figure 3D**).

Further subgroup analyses were conducted according to age, sex, and time from onset to admission after PSM. The administration of GCs demonstrated no notable correlation with CFR in any subgroup (see **Supplementary Figures 3A, B**). However, the administration of GCs was associated with an elevated risk of HAI in patients both with mild and severe SFTS (p = 0.024, p = 0.015, see **Supplementary Figures 3C, D**).

### 3.5 Effect of GCs regimens on patients with SFTS

GCs therapy appeared to reduce mortality in severe patients, but this effect became insignificant after PSM. Therefore, we further investigated the effect of GCs regimen on the CFR and HAI in patients with SFTS, including the timing, duration and dose of GCs treatment. The use of high-dose GCs was associated with an increased CFR (p = 0.001, OR = 3.693, 95% CI: 1.710-8.345); especially in mild patients (p = 0.018, OR = 13.167, 95% CI: 2.195-251.087). Early use of GCs was also associated with increased CFR (p = 0.035, OR = 2.292, 95% CI: 1.071-5.066); especially in severe patients (p = 0.050, OR = 2.868, 95% CI: 1.021-8.546) (**Table 2**). The timing, duration, and dose of GCs therapy did not have a significant effect on HAI (see **Supplementary Table 4**).

**Table 2:** Effect of GC regime on CFR in patients with SFTS after propensity score matching.

Severity of SFTS	Variables	Levels	CFR (%)	p-Value	OR (95%CI)
All patients (n =303)	Initiation of GC therapy	> 6 days	12 (6.78%)	0.035	–
		≤ 6 days	18 (14.29%)		2.292 (1.071, 5.066)
	Duration of GC therapy	> 3 days	14 (10.69%)	0.690	1.167 (0.541, 2.489)
		≤ 3 days	16 (9.3%)		–
	Dose of GC therapy	high	19 (17.92%)	0.001	3.693 (1.710, 8.345)
		low	11 (5.58%)		–
Mild patients (n=237)	Initiation of GC therapy	> 6 days	4 (2.8%)	0.861	–
		≤ 6 days	3 (3.19%)		1.146 (0.221, 5.312)
	Duration of GC therapy	> 3 days	5 (5.26%)	0.109	3.889 (0.819, 27.559)
		≤ 3 days	2 (1.41%)		–
	Dose of GC therapy	high	6 (7.69%)	0.018	13.167 (2.195, 251.087)
		low	1 (0.63%)		–
Severe patients (n=66)	Initiation of GC therapy	> 6 days	8 (23.53%)	0.050	–
		≤ 6 days	15 (46.88%)		2.868 (1.021, 8.546)
	Duration of GC therapy	> 3 days	9 (25%)	0.069	–
		≤ 3 days	14 (46.67%)		2.625 (0.941, 7.661)
	Dose of GC therapy	high	13 (46.43%)	0.093	2.427 (0.870, 7.002)
		low	10 (26.32%)		–

GC doses were calculated as dexamethasone equivalence, with > 5 mg/d defined as high dose and ≤ 5 mg/d defined as moderate dose.

GC, glucocorticoid; CFR, case fatality rate; OR, odds ratio; CI, confidence interval.

**Figure 4:**
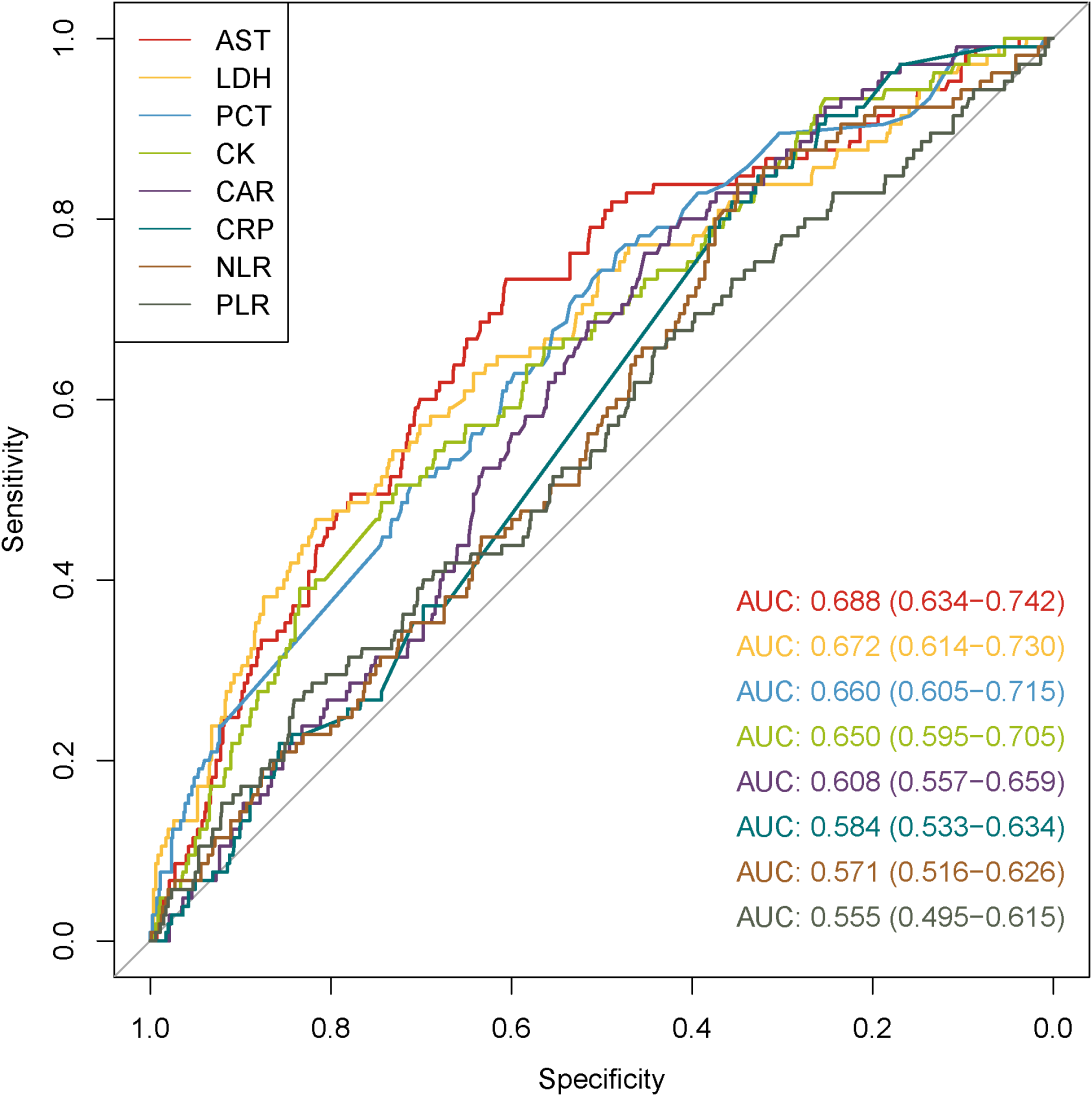
ROC curves of inflammatory and organ injury biomarkers for predicting fatal outcomes in patients with SFTS.

**Figure 5:**
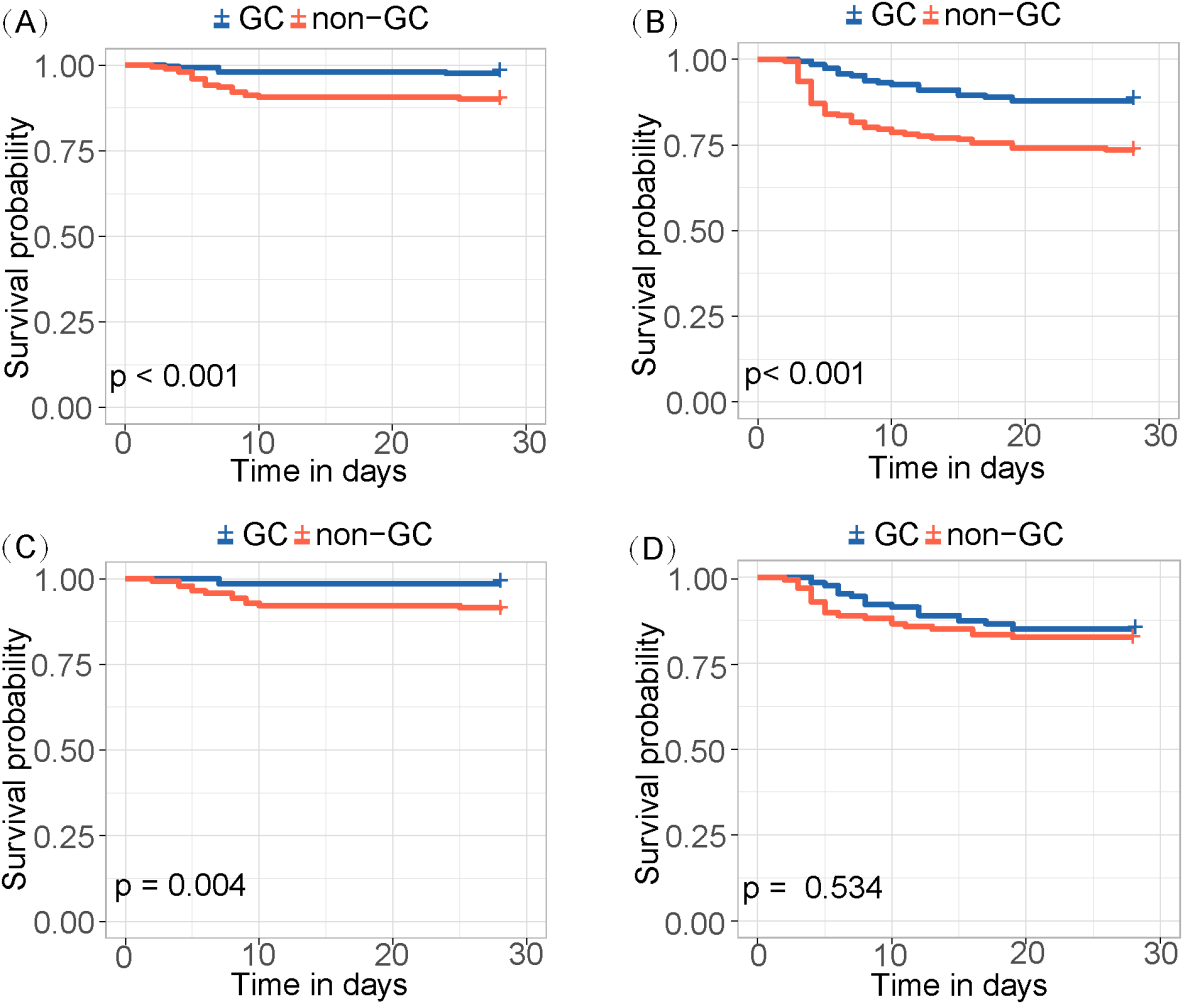
Kaplan-Meier survival curves for **(A)** low-level AST and **(B)** high-level AST groups before PSM. Kaplan-Meier survival curves for **(C)** low-level AST and **(D)** high-level AST groups after PSM.

ROCs were depicted to evaluate the predictive capabilities of PCT, CAR, CRP, NLR, PLR as biomarkers of inflammation and AST, LDH, CK as organ injury biomarkers (**Figure 4**). CRP, NLR, and PLR showed poor predictive performances (p values of 0.125, 0.193, and 0.401, respectively). The AUCs for AST, LDH, PCT, CK and CAR were 0.688 (95% CI: 0.634-0.742, p < 0.001), 0.672 (95% CI: 0.614-0.730, p < 0.001), 0.660 (95% CI: 0.605-0.715, p = 0.001), 0.650 (95% CI: 0.595-0.705, p = 0.001), and 0.608 (95% CI: 0.557-0.659, p = 0.032), respectively. The maximum Youden index identified 189.75 U/L as the optimal critical value of AST, at which its sensitivity and specificity reached 0.733 and 0.606, respectively. Then, the patients were divided into low and high AST groups based on this value. GCs therapy reduced the mortality in both the low and high AST groups (p < 0.001) (**Figures 5A, B**). After PSM, this effect remained only in the low AST group (p = 0.004, **Figure 5C**).

## 4 Discussion

In this multicenter retrospective analysis, we identified older age, consciousness disturbance, decreased monocyte counts, prolonged APTT, and high viral load as significant risk factors for fatal outcomes in SFTS patients. Based on these factors, the new nomogram showed a good prognostic performance, with an AUC of 0.908 (95% CI: 0.879-0.936), a sensitivity of 83.2%, and a specificity of 76.7%.

Older age has been recognized as a risk factor for serious and fatal outcomes of SFTS in previous studies (Li et al., 2018; Zhong et al., 2024). Neurologic symptoms have long been associated with the severity of SFTS outcomes, as evidenced by the mortality exceeds 40% in patients with SFTS-related encephalopathy (Xu et al., 2021). In our study, the results of multivariate analysis showed that consciousness disturbance was a strong predictor of death (p<0.001, aOR 12.994, 95% CI: 7.625-22.142). Neurologic symptoms have been a major component of prognostic models developed by Wang et al. and Xia et al (Wang et al., 2021; Xia et al., 2023). Therefore, the consciousness of SFTS patients should be fully evaluated for predicting their prognoses. SFTSV attacks the central nervous system through such mechanisms as direct invasion, cytokine storms, and immune dysregulation (Shan et al., 2024). It has been proposed that peripheral monocytes may be a key cell type affected by SFTS, and the results of this study suggest that acute SFTSV infection leads to significant loss of monocyte subsets and impaired monocyte function, and that the involvement of monocytes in the pathogenesis of SFTS is through the mechanism of disturbed innate immune response (Peng et al., 2016). Significant elevation in viral load can induce excessive release of pro-inflammatory cytokines that arouse a storm to cause severe inflammatory responses and widespread tissue and organ damage (He et al., 2021). Coagulation dysfunction is a common event in patients with SFTS, as shown by a significant prolongation of APTT observed in fatal patients compared to survivors. SFTSV infection results in substantial endothelial damage, which ends up with DIC and hemorrhage in vital tissues and organs (Wang et al., 2022a). In the present study, the predictive nomogram incorporated clinical, virological, and laboratory variables, thereby achieving an accuracy higher than its precedents.

Cytokine storm has been demonstrated to correlate with the severity of SFTS. Consequently, we proceeded to evaluate the efficacy of GCs as an anti-inflammatory intervention. It has been reported that the addition of GCs may be beneficial for treating acute encephalopathy and myocardial dysfunction after SFTSV infection (Nakamura et al., 2018). However, several retrospective studies from China, Korea, and Japan have shown that GCs use may increase the risk of mortality and secondary infections in SFTS patients (Jung et al., 2021; Kawaguchi et al., 2021; Xiong et al., 2022). A recent study shows that patients with severe SFTS may benefit from low to moderate doses of GCs, whereas in patients with mild SFTS, GCs treatment is significantly associated with increased CFR (Wang et al., 2023). Therefore, the efficacy of GCs on SFTS remains controversial, due to methodological limitations in previous studies.

In the present study, we observed that GCs therapy reduced mortality in severe patients but not in mild patients. However, significance disappeared after matching patients’ baseline information. We also verified that early and high-dose GCs use was associated with an elevated risk of mortality. One explanation may be that the host immune response is not sufficient enough to inhibit viral replication during the initial stage, while early utilization of GCs may impede viral clearance (Liu WD. et al., 2024). GCs can inhibit the protective function of T cells and prevent B cells from producing antibodies, thus increasing plasma viral load and impairing host immune function (He et al., 2018). The results of the present study showed that in mild patients, the increased CFR was mainly attributed to high-dose GCs, a finding that is consistent with that of Wang et al (Wang et al., 2023). Previous studies also suggested that GCs therapy was associated with other adverse events, especially secondary bacterial/fungal infections (Jung et al., 2021; Kawaguchi et al., 2021) and hyperglycemia (Cai et al., 2021). Besides, these findings indicated that secondary infections were increased in both severe and mild patients treated with glucocorticoids, and the use of high-dose GCs compared with low-dose therapy was associated with more severe adverse effects, thus further worsening the prognosis, most likely through hyperglycemia and secondary infections. Consequently, early and high-dose use of GCs is ineffective or even harmful in patients with SFTS, especially in mild patients. Also, monitoring is needed for adverse events especially secondary infections that may occur during and after treatment.

The therapeutic efficacy of GCs owes primarily to their anti-inflammatory properties, whereas excessive activation of inflammatory responses represents a significant contributor to multi-organ injury. Therefore, we plotted ROC curves with the most commonly used markers of inflammation and organ injury, including AST, LDH, CK, PCT, CRP, CAR, NLR, and PLR (Wei Y. et al., 2022; Liu Z. et al., 2024). Among them, AST generated the largest AUC. Moreover, AST-stratified analysis indicated that administration of GCs was associated with a reduction in mortality after PSM (p=0.004) in patients with low-level AST (<189.75 U/L). Consistent with the results of the large single-center study by Li et al., elevated AST indicates the presence of acute inflammation and multiorgan damage in the early stage of SFTS (Li et al., 2018). Therefore, the use of GCs before the onset of excessive inflammatory response and multiorgan injury may reduce the mortality of SFTS patients, and AST may be a reliable indicator for predicting the progression of SFTS and determining the start of GCs therapy. In addition, during the use of GCs in patients with SFTS, HAI should be monitored, especially in females and those with an onset-to-admission time of less than 6 days.

However, our study does have certain limitations. First, as a retrospective study, it is inevitably subject to bias. There are currently no standardized protocols for GCs therapy. Usually relying on their clinical experience, physicians often hold confusion about indications and tend to use GCs in severe SFTS cases. Second, due to the absence of cytokines and ferritin-related data, we could not fully explore the anti-inflammatory mechanism of GCs. Third, consciousness disturbance was based on subjective criteria, rather than the Glasgow Coma Scale for quantitative measurement. Last, there are inherent limitations in the prognostic model, as patient mortality rates differ between the two hospitals. Nevertheless, our findings should be validated in an external cohort.

## 5 Conclusions

A new nomogram based on five independent risk factors has demonstrated a strong ability to predict prognosis in SFTS. GCs therapy may reduce mortality in patients with severe SFTS, whereas early use of GCs may increase mortality in patients with severe SFTS and high dose of GCs may increase mortality in patients with mild SFTS. Initiation of GCs therapy in patients with low AST may reduce mortality. In addition, the risk of hospital-acquired infections after treatment with GCs should be monitored in all patients with SFTS.

## Data Availability

The data analyzed in this study is subject to the following licenses/restrictions: The raw data supporting the conclusions of this article will be made available by the authors, without undue reservation. Requests to access these datasets should be directed to ZP, zhihangpeng@njmu.edu.cn.

## References

[B1] Cai J. Li H. Zhang C. Chen Z. Liu H. Lei F.. (2021). The neutrophil-to-lymphocyte ratio determines clinical efficacy of corticosteroid therapy in patients with COVID-19. Cell Metab. 33, 258–269.e3. doi: 10.1016/j.cmet.2021.01.002 33421384 PMC7832609

[B2] Chen G. Chen T. Shu S. N. Ma K. Wang X. J. Wu D. (2022). Expert consensus on diagnosis and treatment of severe fever with thrombocytopenia syndrome. Chin. J. Clin. Infect. Diseases 15, 253–263. doi: 10.3760/cma.j.issn.1674-2397.2022.04.003

[B3] Chinese Society of Infectious Diseases Chinese Medical Association (2022). Consensus on the diagnosis and treatment of severe fever with thrombocytopenia syndrome. Chin. J. Infect. Dis. 40, 711–721. doi: 10.3760/cma.j.cn311365-20221017-00425

[B4] FakhriRavari A. Jin S. Kachouei F. H. Le D. Lopez M. (2021). Systemic corticosteroids for management of COVID-19: Saving lives or causing harm? Int. J. Immunopathol. Pharmacol. 35, 20587384211063976. doi: 10.1177/20587384211063976 34923856 PMC8725047

[B5] Ge H. H. Wang G. Guo P. J. Zhao J. Zhang S. Xu Y. L.. (2022). Coinfections in hospitalized patients with severe fever with thrombocytopenia syndrome: A retrospective study. J. Med. Virol. 94, 5933–5942. doi: 10.1002/jmv.v94.12 36030552

[B6] He Q. Song X. Huang Y. Huang W. Ye B. Luo H.. (2018). Dexamethasone stimulates hepatitis B virus (HBV) replication through autophagy. Med. Sci. Monit. 24, 4617–4624. doi: 10.12659/MSM.906250 29972684 PMC6064191

[B7] He Z. Wang B. Li Y. Hu K. Yi Z. Ma H.. (2021). Changes in peripheral blood cytokines in patients with severe fever with thrombocytopenia syndrome. J. Med. Virol. 93, 4704–4713. doi: 10.1002/jmv.26877 33590892 PMC8360139

[B8] Jia B. Yan X. Chen Y. Wang G. Liu Y. Xu B.. (2017). A scoring model for predicting prognosis of patients with severe fever with thrombocytopenia syndrome. PloS Neglected Trop. Diseases 11, e0005909. doi: 10.1371/journal.pntd.0005909 PMC5626493 28934195

[B9] Jung S. I. Kim Y. E. Yun N. R. Kim C. M. Kim D. M. Han M. A.. (2021). Effects of steroid therapy in patients with severe fever with Thrombocytopenia syndrome: A multicenter clinical cohort study. PloS Negl. Trop. Dis. 15, e0009128. doi: 10.1371/journal.pntd.0009128 33606699 PMC7928499

[B10] Katsurada N. Suzuki M. Aoshima M. Yaegashi M. Ishifuji T. Asoh N.. (2017). The impact of virus infections on pneumonia mortality is complex in adults: a prospective multicentre observational study. BMC Infect. Dis. 17, 755. doi: 10.1186/s12879-017-2858-y 29212450 PMC5719746

[B11] Kawaguchi T. Umekita K. Yamanaka A. Hara S. Yamaguchi T. Inoue E.. (2021). Corticosteroids may have negative effects on the management of patients with severe fever with thrombocytopenia syndrome: A case–control study. Viruses 13, 785. doi: 10.3390/v13050785 33925061 PMC8145003

[B12] Kim E. H. Park S. J. (2023). Emerging tick-borne dabie bandavirus: virology, epidemiology, and prevention. Microorganisms 11, 2309. doi: 10.3390/microorganisms11092309 37764153 PMC10536723

[B13] Li H. Lu Q. B. Xing B. Zhang S. F. Liu K. Du J.. (2018). Epidemiological and clinical features of laboratory-diagnosed severe fever with thrombocytopenia syndrome in China, 2011-17: a prospective observational study. Lancet Infect. Dis. 18, 1127–1137. doi: 10.1016/S1473-3099(18)30293-7 30054190

[B14] Liu Z. Ge Z. Pan W. Zhang R. Jiang Z. Zhao C.. (2024). Development and validation of the PLNA score to predict cytokine storm in acute-phase SFTS patients: A single-center cohort study. Int. Immunopharmacol. 136, 112288. doi: 10.1016/j.intimp.2024.112288 38823181

[B15] Liu W. D. Wang J. T. Shih M. C. Chen K. H. Huang S. T. Huang C. F.. (2024). Effect of early dexamethasone on outcomes of COVID-19: A quasi-experimental study using propensity score matching. J. Microbiol. Immunol. Infect. 57, 414–425. doi: 10.1016/j.jmii.2024.02.002 38402071

[B16] Ministry of Health, PRC (2011). Guideline for prevention and treatment of severe fever with thrombocytopenia syndrome (2010 version). Chin. J. Clin. Infect. Dis. 04, 193–194. doi: 10.3760/cma.j.issn.1674-2397.2011.04.001

[B17] Nakamura S. Azuma M. Maruhashi T. Sogabe K. Sumitani R. Uemura M.. (2018). Steroid pulse therapy in patients with encephalopathy associated with severe fever with thrombocytopenia syndrome. J. Infect. Chemother. 24, 389–392. doi: 10.1016/j.jiac.2017.11.004 29428565

[B18] Peng C. Wang H. Zhang W. Zheng X. Tong Q. Jie S.. (2016). Decreased monocyte subsets and TLR4-mediated functions in patients with acute severe fever with thrombocytopenia syndrome (SFTS). Int. J. Infect. Diseases 43, 37–42. doi: 10.1016/j.ijid.2015.12.009 26701820

[B19] Riley R. D. Ensor J. Snell K. I. E. Harrell F. E. Martin G. P. Reitsma J. B.. (2020). Calculating the sample size required for developing a clinical prediction model. BMJ 368, m441. doi: 10.1136/bmj.m441 32188600

[B20] Shan D. Chen W. Liu G. Zhang H. Chai S. Zhang Y. (2024). Severe fever with thrombocytopenia syndrome with central nervous system symptom onset: a case report and literature review. BMC Neurol. 24, 158. doi: 10.1186/s12883-024-03664-6 38730325 PMC11084135

[B21] Shang L. Zhao J. Hu Y. Du R. Cao B. (2020). On the use of corticosteroids for 2019-nCoV pneumonia. Lancet 395, 683–684. doi: 10.1016/S0140-6736(20)30361-5 32122468 PMC7159292

[B22] Wang B. He Z. Yi Z. Yuan C. Suo W. Pei S.. (2021). Application of a decision tree model in the early identification of severe patients with severe fever with thrombocytopenia syndrome. PloS One 16, e0255033. doi: 10.1371/journal.pone.0255033 34329338 PMC8324211

[B23] Wang Y. Song Z. Wei X. Yuan H. Xu X. Liang H.. (2022a). Clinical laboratory parameters and fatality of Severe fever with thrombocytopenia syndrome patients: A systematic review and meta-analysis. PloS Negl. Trop. Dis. 16, e0010489. doi: 10.1371/journal.pntd.0010489 35714138 PMC9246219

[B24] Wang Y. Song Z. Xu X. Wei X. Yuan H. Liang H.. (2022b). Clinical symptoms associated with fatality of severe fever with thrombocytopenia syndrome: A systematic review and meta-analysis. Acta Trop. 232, 106481. doi: 10.1016/j.actatropica.2022.106481 35461803

[B25] Wang W. Wang Z. Chen Z. Liang M. Zhang A. Sheng H.. (2024). Construction of an early differentiation diagnosis model for patients with severe fever with thrombocytopenia syndrome and hemorrhagic fever with renal syndrome. J. Med. Virol. 96, e29626. doi: 10.1002/jmv.29626 38654664

[B26] Wang G. Xu Y. L. Zhu Y. Yue M. Zhao J. Ge H. H.. (2023). Clinical efficacy of low-dose glucocorticoid therapy for critically ill patients with severe fever with thrombocytopenia syndrome: A retrospective cohort study. Int. J. Infect. Dis. 130, 153–160. doi: 10.1016/j.ijid.2023.03.015 36921682

[B27] Wei X. Tu L. Qiu L. Chen M. Wang Y. Du M.. (2022). Scoring model for predicting the occurrence of severe illness in hospitalized patients with severe fever with thrombocytopenia syndrome. Jpn. J. Infect. Dis. 75, 382–387. doi: 10.7883/yoken.JJID.2021.716 35095026

[B28] Wei Y. Wang Z. Kang L. He L. Sheng N. Qin J.. (2022). NLR, A convenient early-warning biomarker of fatal outcome in patients with severe fever with thrombocytopenia syndrome. Front. Microbiol. 13, 907888. doi: 10.3389/fmicb.2022.907888 35814714 PMC9262381

[B29] Xia G. Sun S. Zhou S. Li L. Li X. Zou G.. (2023). A new model for predicting the outcome and effectiveness of drug therapy in patients with severe fever with thrombocytopenia syndrome: A multicenter Chinese study. PloS Neglected Trop. Diseases 17, e0011158. doi: 10.1371/journal.pntd.0011158 PMC10019728 36877734

[B30] Xiong L. Xu L. Lv X. Zheng X. (2022). Effects of corticosteroid treatment in patients with severe fever with thrombocytopenia syndrome: A single-center retrospective cohort study. Int. J. Infect. Dis. 122, 1026–1033. doi: 10.1016/j.ijid.2022.07.001 35803466

[B31] Xu Y. Shao M. Liu N. Dong D. Tang J. Gu Q. (2021). Clinical feature of severe fever with thrombocytopenia syndrome (SFTS)-associated encephalitis/encephalopathy: a retrospective study. BMC Infect. Dis. 21, 904. doi: 10.1186/s12879-021-06627-1 34479504 PMC8418043

[B32] Yu X. J. Liang M. F. Zhang S. Y. Liu Y. Li J. D. Sun Y. L.. (2011). Fever with thrombocytopenia associated with a novel bunyavirus in China. N. Engl. J. Med. 364, 1523–1532. doi: 10.1056/NEJMoa1010095 21410387 PMC3113718

[B33] Zhong F. Lin X. Zheng C. Tang S. Yin Y. Wang K.. (2024). Establishment and validation of a clinical risk scoring model to predict fatal risk in SFTS hospitalized patients. BMC Infect. Diseases 24, 975. doi: 10.1186/s12879-024-09898-6 39272027 PMC11401407

