# Supplementary material for: Fatal risk factors and the efficacy of glucocorticoid therapy in severe fever with thrombocytopenia syndrome: a multicenter retrospective cohort study
Source: Front Cell Infect Microbiol. 2025 May 8;15:1531880. doi: 10.3389/fcimb.2025.1531880 (PMC12095230; doi:10.3389/fcimb.2025.1531880)
Supplement: Supplementary file 1 [file Table1.docx]

**
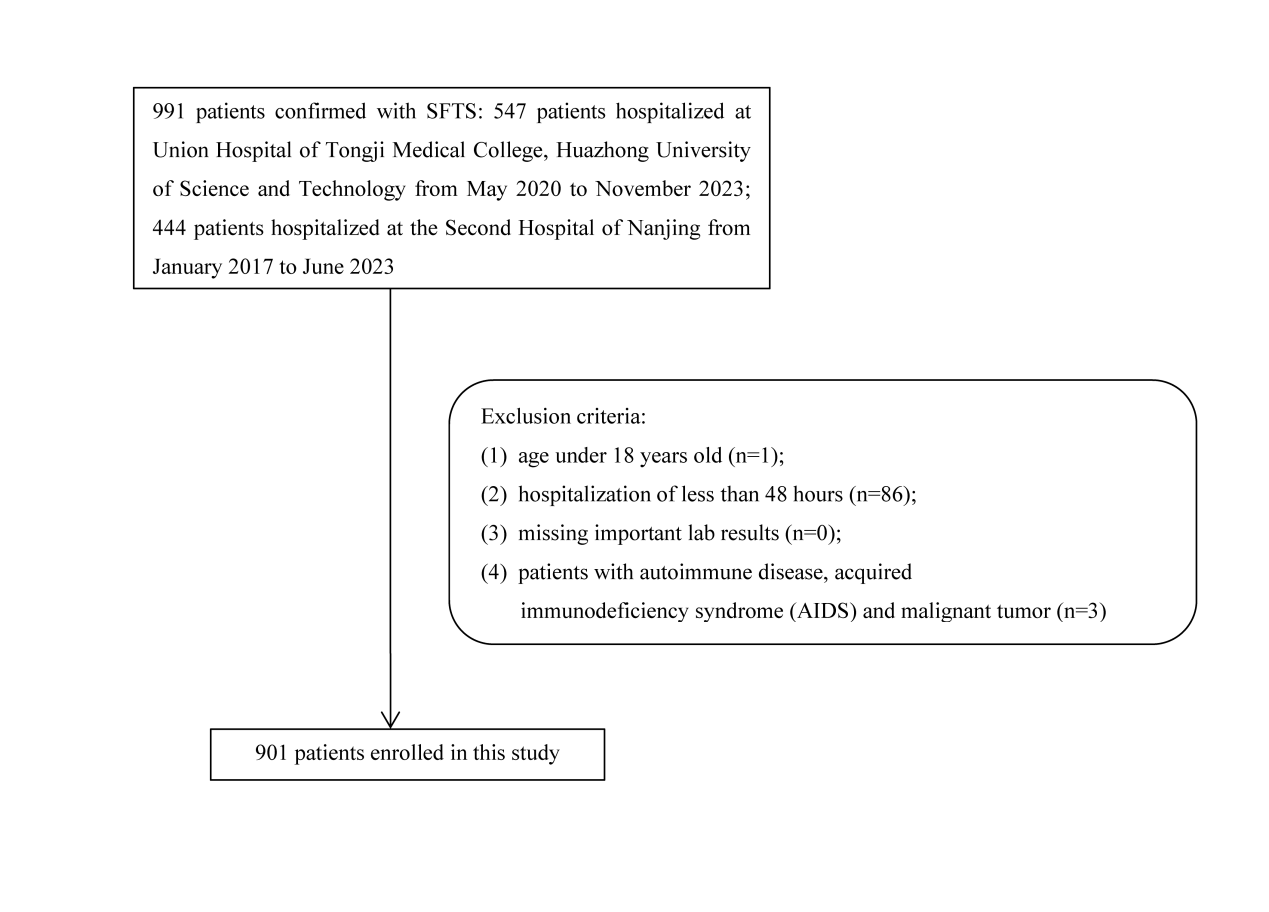
**

Figure S1. Flow chart of the study design.

**
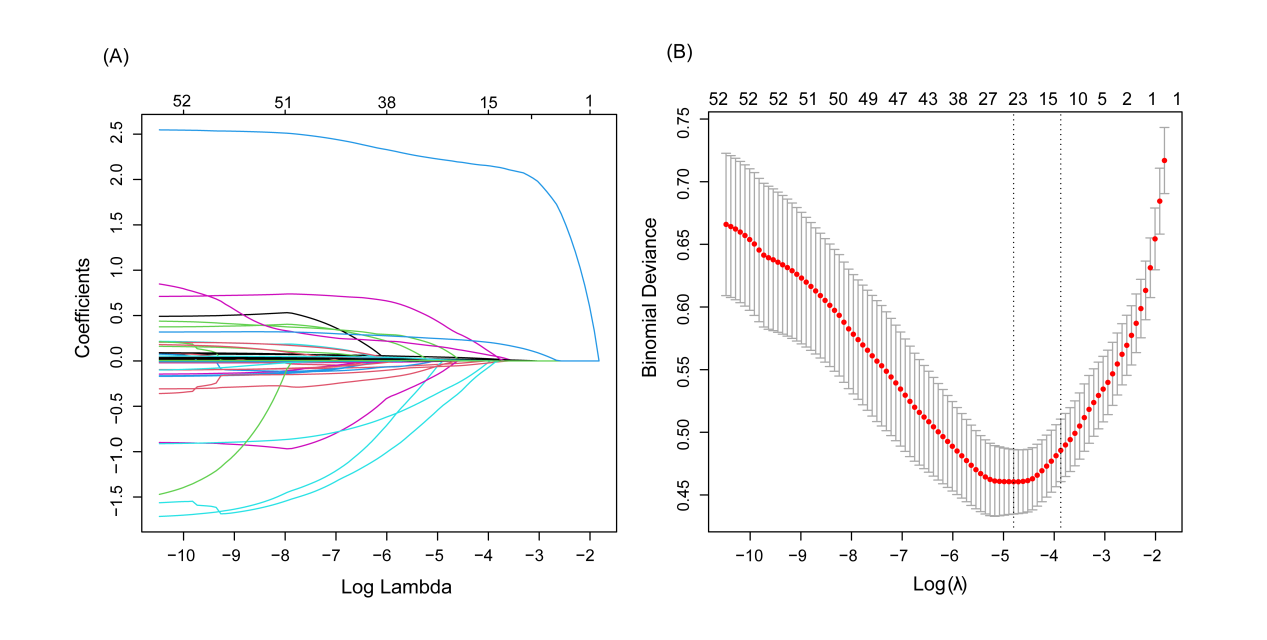
**

Figure S2. Screening of risk factors for fatal outcome in patients with SFTS based on Lasso regression.

**
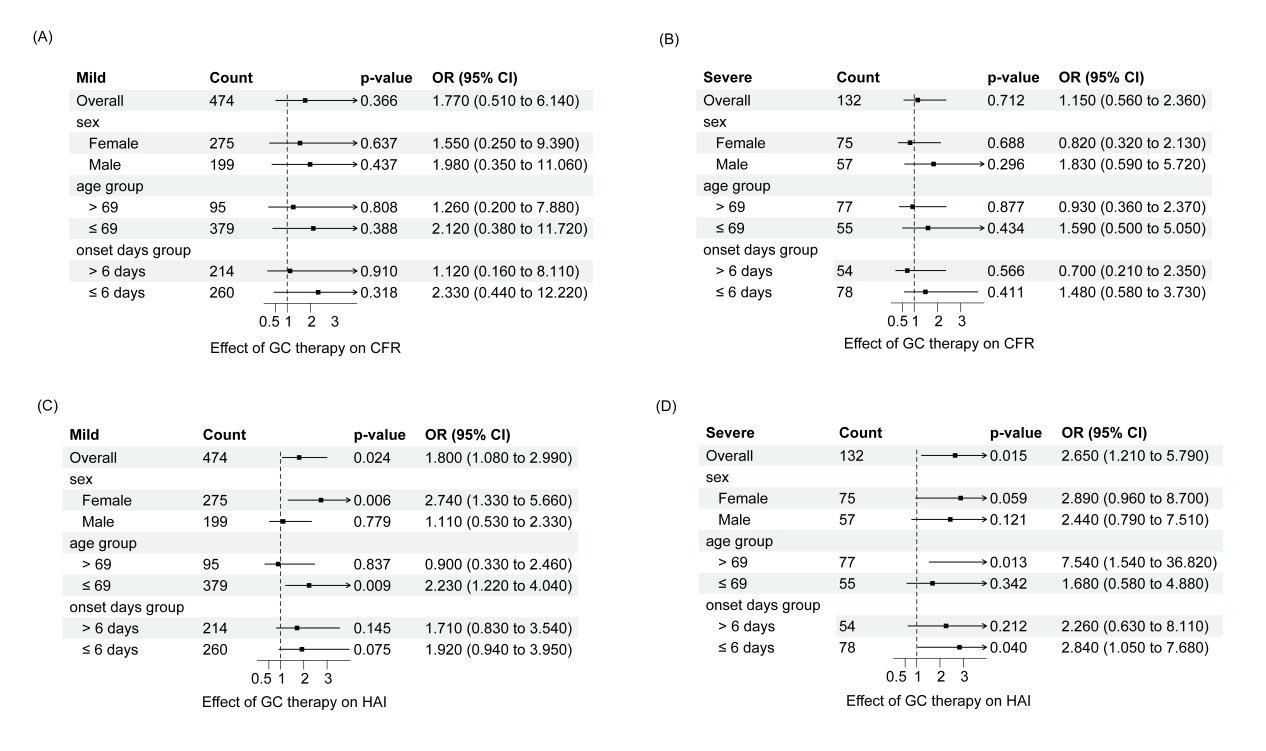
**

Figure S3. Evaluation of the effect of GC therapy on CFR and HAI.

(A) Effect of GC therapy on CFR in patients with mild-SFTS; (B) Effect of GC therapy on CFR in patients with severe-SFTS; (C) Effect of GC therapy on HAI in patients with mild-SFTS; (D) Effect of GC therapy on HAI in patients with severe-SFTS.

Datapoints show OR (odds ratio) and error bars show 95% CI (confidence interval).

Table S1 Comparison of clinical characteristics and laboratory results of patients with SFTS in the nonfatal and fatal groups.

| Variables | Total (n = 901) | Nonfatal (n = 796) | Fatal (n = 105) | p-Value |
| --- | --- | --- | --- | --- |
| Age (years) | 64.00 (56.00, 70.00) | 63.00 (56.00, 70.00) | 70.00 (60.00, 75.00) | <0.001 |
| Time from onset to admission (days) | 6.00 (4.00, 7.00) | 6.00 (5.00, 7.00) | 5.00 (4.00, 7.00) | 0.005 |
| Male, n (%) | 395 (43.8) | 345 (43.3) | 50 (47.6) | 0.468 |
| Hypertension, n (%) | 250 (27.7) | 213 (26.8) | 37 (35.2) | 0.088 |
| Diabetes, n (%) | 103 (11.4) | 93 (11.7) | 10 (9.5) | 0.624 |
| CHD, n (%) | 32 (3.6) | 29 (3.6) | 3 (2.9) | 0.898 |
| Systolic blood pressure (mmHg) | 119.00 (107.00, 130.00) | 118.00 (107.00, 130.00) | 120.00 (107.00, 130.00) | 0.803 |
| Respiratory rate (breaths/min) | 20.00 (19.00, 20.00) | 20.00 (19.00, 20.00) | 20.00 (19.00, 20.00) | 0.009 |
| Fever, n (%) | 887 (98.4) | 785 (98.6) | 102 (97.1) | 0.466 |
| Headache, n (%) | 155 (17.2) | 139 (17.5) | 16 (15.2) | 0.667 |
| Consciousness disturbance, n (%) | 167 (18.5) | 91 (11.4) | 76 (72.4) | <0.001 |
| Lymphadenopathy, n (%) | 98 (10.9) | 86 (10.8) | 12 (11.4) | 0.979 |
| Cough, n (%) | 199 (22.1) | 173 (21.7) | 26 (24.8) | 0.563 |
| Phlegm, n (%) | 145 (16.1) | 123 (15.5) | 22 (21.0) | 0.193 |
| Vomiting, n (%) | 328 (36.4) | 285 (35.8) | 43 (41.0) | 0.356 |
| Abdominal pain, n (%) | 94 (10.4) | 80 (10.1) | 14 (13.3) | 0.387 |
| Diarrhea, n (%) | 371 (41.2) | 318 (39.9) | 53 (50.5) | 0.051 |
| Muscle pain, n (%) | 242 (26.9) | 227 (28.5) | 15 (14.3) | 0.003 |
| Bleeding, n (%) | 116 (12.9) | 85 (10.7) | 31 (29.5) | <0.001 |
| Coinfection, n (%) | 503 (55.8) | 428 (53.8) | 75 (71.4) | 0.001 |
| GCs, n (%) | 405 (45.0) | 330 (41.5) | 75 (71.4) | ＜0.001 |
| Ribavirin, n (%) | 699 (77.6) | 603 (75.8) | 96 (91.4) | <0.001 |
| Immunoglobulin, n (%) | 524 (58.2) | 437 (54.9) | 87 (82.9) | <0.001 |
| RBC (10^9^/L) | 4.25 (3.88, 4.63) | 4.24 (3.88, 4.62) | 4.27 (3.99, 4.68) | 0.108 |
| HGB (g/L) | 128.00 (117.00, 141.00) | 128.00 (116.75, 141.00) | 131.00 (121.00, 145.00) | 0.038 |
| WBC (10^9^/L) | 3.12 (1.91, 5.53) | 3.21 (1.99, 5.66) | 2.33 (1.64, 4.11) | 0.001 |
| Neutrophils (10^9^/L) | 1.92 (1.10, 4.07) | 2.00 (1.12, 4.12) | 1.62 (1.03, 3.32) | 0.092 |
| Lymphocytes (10^9^/L) | 0.64 (0.43, 1.05) | 0.67 (0.46, 1.11) | 0.44 (0.31, 0.71) | <0.001 |
| Monocytes (10^9^/L) | 0.15 (0.07, 0.34) | 0.16 (0.09, 0.37) | 0.07 (0.05, 0.13) | <0.001 |
| PLT (10^9^/L) | 50.00 (35.00, 69.00) | 52.00 (36.00, 71.00) | 38.00 (29.00, 49.00) | <0.001 |
| TBIL (umol/L) | 9.10 (6.70, 12.30) | 9.15 (6.68, 12.22) | 8.80 (6.80, 12.50) | 0.850 |
| DBIL (umol/L) | 4.20 (3.10, 6.10) | 4.20 (3.10, 6.10) | 4.20 (2.90, 6.30) | 0.988 |
| ALT (U/L) | 69.00 (41.70, 115.00) | 66.00 (41.18, 108.25) | 92.00 (50.00, 150.70) | <0.001 |
| AST (U/L) | 165.00 (86.40, 310.00) | 146.00 (84.35, 280.35) | 270.90 (166.80, 570.00) | <0.001 |
| ALP (U/L) | 68.00 (55.00, 89.00) | 68.00 (54.00, 89.00) | 69.00 (56.00, 91.00) | 0.852 |
| GGT (U/L) | 36.50 (22.00, 76.00) | 36.00 (22.00, 76.00) | 43.00 (26.00, 88.00) | 0.083 |
| ALB (U/L) | 33.20 (30.10, 36.40) | 33.30 (30.28, 36.50) | 32.70 (29.70, 35.40) | 0.050 |
| GLOB (g/L) | 25.50 (22.90, 28.70) | 25.45 (22.80, 28.40) | 26.80 (23.80, 31.20) | 0.006 |
| SCr (umol/L) | 71.70 (59.70, 89.00) | 70.00 (58.80, 85.12) | 89.00 (69.10, 123.80) | <0.001 |
| BUN (mmol/L) | 5.17 (3.70, 7.00) | 5.00 (3.58, 6.67) | 6.90 (5.10, 10.60) | <0.001 |
| LDH (U/L) | 692.00 (433.00, 1114.00) | 649.00 (417.00, 1023.25) | 1024.00 (633.00, 1767.00) | <0.001 |
| CK (U/L) | 512.00 (207.00, 1125.29) | 483.00 (188.75, 1119.82) | 1044.00 (343.00, 1937.00) | <0.001 |
| Na (mmol/L) | 135.20 (132.00, 138.00) | 135.20 (132.00, 138.10) | 135.00 (132.00, 137.90) | 0.554 |
| K (mmol/L) | 3.67 (3.32, 3.98) | 3.63 (3.32, 3.94) | 3.81 (3.45, 4.10) | 0.006 |
| Ca (mmol/L) | 1.96 (1.87, 2.04) | 1.96 (1.88, 2.05) | 1.90 (1.79, 2.00) | <0.001 |
| D-Dimer (mg/L) | 3.05 (1.42, 5.14) | 2.82 (1.35, 5.14) | 4.90 (2.58, 9.09) | <0.001 |
| PT (s) | 12.20 (11.20, 13.00) | 12.20 (11.20, 12.93) | 12.60 (11.40, 13.30) | 0.044 |
| INR | 0.99 (0.92, 1.06) | 0.98 (0.91, 1.04) | 1.07 (0.98, 1.14) | <0.001 |
| APTT (s) | 45.90 (38.20, 55.30) | 44.95 (38.00, 54.20) | 53.40 (43.70, 66.70) | <0.001 |
| FIB (g/L) | 2.44 (2.07, 2.83) | 2.48 (2.12, 2.86) | 2.14 (1.75, 2.49) | <0.001 |
| TT (s) | 23.60 (19.80, 30.60) | 23.45 (19.80, 29.40) | 27.10 (20.30, 39.70) | 0.006 |
| CRP (mg/L) | 9.00 (4.06, 11.49) | 9.00 (3.78, 11.40) | 9.00 (9.00, 13.60) | 0.005 |
| PCT (ng/L) | 0.32 (0.10, 1.05) | 0.29 (0.10, 1.05) | 0.80 (0.27, 1.05) | <0.001 |
| NLR | 2.98 (1.52, 6.27) | 2.94 (1.47, 6.16) | 3.36 (2.14, 6.87) | 0.018 |
| CAR | 0.25 (0.13, 0.36) | 0.25 (0.12, 0.36) | 0.28 (0.24, 0.42) | <0.001 |
| PLR | 74.23 (43.67, 125.49) | 73.36 (43.34, 124.26) | 83.33 (52.94, 153.23) | 0.067 |
| Lg viral load (copies/ml) | 3.80 (2.00, 6.38) | 3.58 (2.00, 5.83) | 6.58 (5.10, 7.25) | <0.001 |

Abbreviations: CHD, coronary heart disease; GCs, glucocorticoids; RBC, red blood cell; HGB, hemoglobin; WBC, white blood cell; PLT, platelet count; TBIL, total bilirubin; DBIL, direct bilirubin; ALT, alanine aminotransaminase; AST, aspartate aminotransferase; ALP, alkaline phosphatase; GGT, γ-glutamyl transferase; ALB, albumin; GLOB, globulin; SCr, serum creatinine; BUN, blood urea nitrogen; LDH, lactate dehydrogenase; CK, creatine phosphokinase; Na, sodium; K, kalium; Ca, calcium; PT, prothrombin time; INR, international normalized ratio; APTT, activated partial thromboplastin time; FIB, fibrinogen; TT, thrombin time; CRP, C-reactive protein; PCT, procalcitonin; NLR, neutrophil-to-lymphocyte ratio; CAR, ratio of C-reactive protein to albumin; PLR, platelet-to-lymphocyte ratio; Lg viral load, log-transformed viral load.

Table S2 Comparison of baseline characteristics between GC and non-GC groups.

| Variables | Total (n = 901) | No GC (n = 496) | GC (n = 405) | p-Value |
| --- | --- | --- | --- | --- |
| Status, n (%) | 105 (11.7) | 30 (6.0) | 75 (18.5) | <0.001 |
| Age (years) | 64.00 (56.00, 70.00) | 64.00 (56.00, 70.00) | 65.00 (56.00, 71.00) | 0.100 |
| Time from onset to admission (days) | 6.00 (4.00, 7.00) | 7.00 (5.00, 7.00) | 6.00 (4.00, 7.00) | 0.027 |
| Male, n (%) | 395 (43.8) | 213 (42.9) | 182 (44.9) | 0.594 |
| Hypertension, n (%) | 250 (27.7) | 137 (27.6) | 113 (27.9) | 0.985 |
| Diabetes, n (%) | 103 (11.4) | 61 (12.3) | 42 (10.4) | 0.424 |
| CHD, n (%) | 32 (3.6) | 18 (3.6) | 14 (3.5) | 1.000 |
| Systolic blood pressure (mmHg) | 119.00 (107.00, 130.00) | 118.00 (107.00, 131.00) | 120.00 (107.00, 129.00) | 0.615 |
| Respiratory rate (breaths/min) | 20.00 (19.00, 20.00) | 20.00 (19.00, 20.00) | 20.00 (19.00, 20.00) | 0.067 |
| Fever, n (%) | 887 (98.4) | 488 (98.4) | 399 (98.5) | 1.000 |
| Headache, n (%) | 155 (17.2) | 98 (19.8) | 57 (14.1) | 0.031 |
| Consciousness disturbance, n (%) | 167 (18.5) | 54 (10.9) | 113 (27.9) | <0.001 |
| Lymphadenopathy, n (%) | 98 (10.9) | 49 ( 9.9) | 49 (12.1) | 0.339 |
| Cough, n (%) | 199 (22.1) | 102 (20.6) | 97 (24.0) | 0.255 |
| Phlegm, n (%) | 145 (16.1) | 76 (15.3) | 69 (17.0) | 0.545 |
| Vomiting, n (%) | 328 (36.4) | 176 (35.5) | 152 (37.5) | 0.572 |
| Abdominal pain, n (%) | 94 (10.4) | 54 (10.9) | 40 ( 9.9) | 0.701 |
| Diarrhea, n (%) | 371 (41.2) | 199 (40.1) | 172 (42.5) | 0.519 |
| Musclepain, n (%) | 242 (26.9) | 135 (27.2) | 107 (26.4) | 0.847 |
| Bleeding, n (%) | 116 (12.9) | 47 ( 9.5) | 69 (17.0) | 0.001 |
| Coinfection, n (%) | 503 (55.8) | 248 (50.0) | 255 (63.0) | <0.001 |
| Ribavirin, n(%) | 699 (77.6) | 380 (76.6) | 319 (78.8) | 0.490 |
| immunoglobulin, n(%) | 524 (58.2) | 218 (44.0) | 306 (75.6) | <0.001 |
| RBC (×10^9^/L) | 4.25 (3.88, 4.63) | 4.23 (3.86, 4.61) | 4.26 (3.91, 4.66) | 0.190 |
| HGB (g/L) | 128.00 (117.00, 141.00) | 127.00 (116.00, 141.00) | 129.00 (118.00, 141.00) | 0.213 |
| WBC (×10^9^/L) | 3.12 (1.91, 5.53) | 3.12 (1.98, 5.62) | 3.11 (1.81, 5.35) | 0.349 |
| Neutrophils (%) | 1.92 (1.10, 4.07) | 1.85 (1.09, 3.96) | 2.05 (1.14, 4.13) | 0.376 |
| Lymphocytes (%) | 0.64 (0.43, 1.05) | 0.74 (0.48, 1.18) | 0.56 (0.39, 0.86) | <0.001 |
| Monocytes (%) | 0.15 (0.07, 0.34) | 0.17 (0.09, 0.39) | 0.12 (0.07, 0.26) | <0.001 |
| PLT (×10^9^/L) | 50.00 (35.00, 69.00) | 53.00 (37.00, 74.00) | 46.00 (32.00, 63.00) | <0.001 |
| TBIL (umol/L) | 9.10 (6.70, 12.30) | 9.35 (6.90, 12.62) | 8.70 (6.60, 11.80) | 0.070 |
| DBIL (umol/L) | 4.20 (3.10, 6.10) | 4.30 (3.08, 6.10) | 4.10 (3.10, 6.00) | 0.346 |
| ALT (U/L) | 69.00 (41.70, 115.00) | 66.95 (41.15, 111.50) | 72.00 (44.00, 118.00) | 0.219 |
| AST (U/L) | 165.00 (86.40, 310.00) | 138.90 (76.90, 274.50) | 187.00 (102.00, 364.00) | <0.001 |
| ALP (U/L) | 68.00 (55.00, 89.00) | 67.00 (55.00, 87.00) | 69.00 (54.00, 91.00) | 0.432 |
| GGT (U/L) | 36.50 (22.00, 76.00) | 36.75 (22.00, 79.06) | 36.10 (23.00, 72.00) | 0.810 |
| ALB (U/L) | 33.20 (30.10, 36.40) | 33.15 (30.20, 36.42) | 33.30 (30.00, 36.30) | 0.890 |
| GLOB (g/L) | 25.50 (22.90, 28.70) | 25.60 (23.20, 28.70) | 25.40 (22.80, 28.60) | 0.683 |
| SCr (umol/L) | 71.70 (59.70, 89.00) | 68.40 (57.15, 84.08) | 75.00 (62.00, 95.00) | <0.001 |
| BUN (mmol/L) | 5.17 (3.70, 7.00) | 4.70 (3.40, 6.43) | 5.62 (4.10, 7.89) | <0.001 |
| LDH (U/L) | 692.00 (433.00, 1114.00) | 599.50 (385.75, 940.00) | 820.00 (510.00, 1322.00) | <0.001 |
| CK (U/L) | 512.00 (207.00, 1125.29) | 417.50 (165.75, 1099.75) | 593.00 (270.00, 1196.00) | <0.001 |
| Na (mmol/L) | 135.20 (132.00, 138.00) | 135.70 (133.00, 138.30) | 134.40 (131.70, 137.60) | 0.001 |
| K (mmol/L) | 3.67 (3.32, 3.98) | 3.60 (3.32, 3.92) | 3.72 (3.34, 4.08) | 0.001 |
| Ca (mmol/L) | 1.96 (1.87, 2.04) | 1.97 (1.88, 2.06) | 1.93 (1.84, 2.02) | <0.001 |
| D-Dimer (mg/L) | 3.05 (1.42, 5.14) | 2.44 (1.19, 5.14) | 4.00 (1.76, 6.06) | <0.001 |
| PT (s) | 12.20 (11.20, 13.00) | 12.20 (11.20, 13.00) | 12.20 (11.20, 13.00) | 0.856 |
| INR | 0.99 (0.92, 1.06) | 0.98 (0.92, 1.04) | 0.99 (0.93, 1.08) | 0.020 |
| APTT (s) | 45.90 (38.20, 55.30) | 43.20 (36.80, 52.60) | 48.20 (40.40, 58.10) | <0.001 |
| FIB (g/L) | 2.44 (2.07, 2.83) | 2.50 (2.17, 2.90) | 2.34 (1.98, 2.72) | <0.001 |
| TT (s) | 23.60 (19.80, 30.60) | 23.00 (19.28, 29.10) | 24.50 (20.50, 33.70) | <0.001 |
| CRP (mg/L) | 9.00 (4.06, 11.49) | 9.00 (3.40, 12.26) | 9.00 (5.50, 11.00) | 0.246 |
| PCT (ng/L) | 0.32 (0.10, 1.05) | 0.29 (0.10, 1.05) | 0.36 (0.13, 1.05) | 0.013 |
| NLR | 2.98 (1.52, 6.27) | 2.52 (1.38, 5.33) | 3.62 (1.98, 7.31) | <0.001 |
| CAR | 0.25 (0.13, 0.36) | 0.25 (0.11, 0.36) | 0.26 (0.16, 0.36) | 0.105 |
| PLR | 74.23 (43.67, 125.49) | 70.44 (42.82, 121.54) | 83.67 (45.90, 130.43) | 0.034 |
| Lg viral load (copies/ml) | 3.80 (2.00, 6.38) | 3.16 (2.00, 5.46) | 4.79 (2.99, 6.92) | <0.001 |

Table S3 Comparison of baseline characteristics between GC and non-GC groups in mild and severe SFTS patients before and after propensity score matching.

| Variables | Mild patients before PSM | | | | Mild patients after PSM | | | | Severe patients before PSM | | | | Severe patients after PSM | | | |
| --- | --- | --- | --- | --- | --- | --- | --- | --- | --- | --- | --- | --- | --- | --- | --- | --- |
|  | Total (n = 679) | No GC (n = 414) | GC (n = 265) | p-Value | Total (n = 474) | No GC (n = 237) | GC (n = 237) | p-Value | Total (n = 222) | No GC (n = 82) | GC (n = 140) | p-Value | Total (n = 132) | No GC (n = 66) | GC (n = 66) | p-Value |
| Status, n (%) | 17 (2.5) | 7 (1.7) | 10 (3.8) | 0.149 | 11 (2.3) | 4 (1.7) | 7 (3.0) | 0.542 | 88 (39.6) | 23 (28.0) | 65 (46.4) | 0.010 | 44 (33.3) | 21 (31.8) | 23 (34.8) | 0.854 |
| Age (years) | 62.00 (55.00, 68.00) | 61.50 (55.00, 68.00) | 62.00 (55.00, 68.00) | 0.924 | 61.00 (55.00, 68.00) | 60.00 (55.00, 68.00) | 62.00 (55.00, 69.00) | 0.294 | 71.00 (62.25, 75.00) | 71.00 (63.00, 75.00) | 70.00 (61.00, 75.00) | 0.53 | 70.00 (63.00, 73.25) | 70.00 (60.50, 74.75) | 70.50 (66.00, 73.00) | 0.982 |
| Time from onset to admission (days) | 7.00 (5.00, 7.00) | 7.00 (5.00, 7.00) | 6.00 (5.00, 7.00) | 0.391 | 6.00 (4.00, 7.00) | 6.00 (4.00, 7.00) | 6.00 (4.00, 7.00) | 0.765 | 6.00 (4.00, 7.00) | 6.00 (5.00, 7.00) | 5.00 (4.00, 7.00) | 0.032 | 6.00 (4.00, 7.00) | 6.00 (5.00, 7.00) | 5.00 (4.00, 7.00) | 0.172 |
| Male, n (%) | 294 (43.3) | 176 (42.5) | 118 (44.5) | 0.661 | 199 (42.0) | 98 (41.4) | 101 (42.6) | 0.852 | 101 (45.5) | 37 (45.1) | 64 (45.7) | 1.000 | 57 (43.2) | 28 (42.4) | 29 (43.9) | 1.000 |
| Hypertension, n (%) | 179 (26.4) | 112 (27.1) | 67 (25.3) | 0.673 | 123 (25.9) | 66 (27.8) | 57 (24.1) | 0.402 | 71 (32.0) | 25 (30.5) | 46 (32.9) | 0.829 | 49 (37.1) | 23 (34.8) | 26 (39.4) | 0.719 |
| Diabetes, n (%) | 78 (11.5) | 51 (12.3) | 27 (10.2) | 0.468 | 59 (12.4) | 34 (14.3) | 25 (10.5) | 0.266 | 25 (11.3) | 10 (12.2) | 15 (10.7) | 0.907 | 17 (12.9) | 8 (12.1) | 9 (13.6) | 1.000 |
| CHD, n (%) | 24 (3.5) | 15 (3.6) | 9 (3.4) | 1.000 | 14 (3.0) | 5 (2.1) | 9 (3.8) | 0.416 | 8 (3.6) | 3 (3.7) | 5 (3.6) | 1.000 | 4 (3.0) | 1 (1.5) | 3 (4.5) | 0.612 |
| Systolic blood pressure (mmHg) | 118.00 (107.00, 129.00) | 118.00 (107.00, 131.00) | 117.00 (107.00, 128.00) | 0.297 | 118.00 (107.00, 129.00) | 118.00 (107.00, 131.00) | 117.00 (107.00, 128.00) | 0.382 | 120.50 (107.00, 131.00) | 117.50 (105.25, 131.00) | 121.00 (107.75, 130.25) | 0.681 | 120.00 (109.00, 131.00) | 117.50 (106.25, 131.00) | 120.50 (110.25, 130.00) | 0.485 |
| Respiratory rate (breaths/min) | 20.00 (19.00, 20.00) | 20.00 (19.00, 20.00) | 20.00 (19.00, 20.00) | 0.302 | 20.00 (19.00, 20.00) | 20.00 (19.00, 20.00) | 20.00 (19.00, 20.00) | 0.877 | 20.00 (19.00, 20.00) | 20.00 (19.00, 20.00) | 20.00 (19.00, 20.00) | 0.450 | 20.00 (19.00, 20.00) | 20.00 (18.25, 20.75) | 20.00 (19.00, 20.00) | 0.476 |
| Fever, n (%) | 671 (98.8) | 410 (99.0) | 261 (98.5) | 0.783 | 469 (98.9) | 236 (99.6) | 233 (98.3) | 0.369 | 216 (97.3) | 78 (95.1) | 138 (98.6) | 0.271 | 129 (97.7) | 64 (97.0) | 65 (98.5) | 1.000 |
| Headache, n (%) | 130 (19.1) | 86 (20.8) | 44 (16.6) | 0.212 | 90 (19.0) | 51 (21.5) | 39 (16.5) | 0.198 | 25 (11.3) | 12 (14.6) | 13 (9.3) | 0.319 | 18 (13.6) | 11 (16.7) | 7 (10.6) | 0.447 |
| Consciousness disturbance, n (%) | 17 (2.5) | 10 (2.4) | 7 (2.6) | 1.000 | 9 (1.9) | 2 (0.8) | 7 (3.0) | 0.178 | 150 (67.6) | 44 (53.7) | 106 (75.7) | 0.001 | 79 (59.8) | 41 (62.1) | 38 (57.6) | 0.723 |
| Lymphadenopathy, n (%) | 79 (11.6) | 39 (9.4) | 40 (15.1) | 0.033 | 62 (13.1) | 28 (11.8) | 34 (14.3) | 0.496 | 19 (8.6) | 10 (12.2) | 9 (6.4) | 0.217 | 13 (9.8) | 7 (10.6) | 6 (9.1) | 1.000 |
| Cough, n (%) | 145 (21.4) | 80 (19.3) | 65 (24.5) | 0.129 | 101 (21.3) | 44 (18.6) | 57 (24.1) | 0.178 | 54 (24.3) | 22 (26.8) | 32 (22.9) | 0.614 | 36 (27.3) | 20 (30.3) | 16 (24.2) | 0.558 |
| Phlegm, n (%) | 101 (14.9) | 57 (13.8) | 44 (16.6) | 0.367 | 66 (13.9) | 27 (11.4) | 39 (16.5) | 0.144 | 44 (19.8) | 19 (23.2) | 25 (17.9) | 0.433 | 29 (22.0) | 18 (27.3) | 11 (16.7) | 0.207 |
| Vomiting, n (%) | 241 (35.5) | 146 (35.3) | 95 (35.8) | 0.942 | 170 (35.9) | 84 (35.4) | 86 (36.3) | 0.924 | 87 (39.2) | 30 (36.6) | 57 (40.7) | 0.641 | 46 (34.8) | 24 (36.4) | 22 (33.3) | 0.855 |
| Abdominal pain, n (%) | 74 (10.9) | 45 (10.9) | 29 (10.9) | 1.000 | 55 (11.6) | 27 (11.4) | 28 (11.8) | 1.000 | 20 (9.0) | 9 (11.0) | 11 (7.9) | 0.589 | 11 (8.3) | 6 (9.1) | 5 (7.6) | 1.000 |
| Diarrhea, n (%) | 265 (39.0) | 159 (38.4) | 106 (40.0) | 0.738 | 200 (42.2) | 103 (43.5) | 97 (40.9) | 0.642 | 106 (47.7) | 40 (48.8) | 66 (47.1) | 0.923 | 62 (47.0) | 34 (51.5) | 28 (42.4) | 0.383 |
| Musclepain, n (%) | 198 (29.2) | 116 (28.0) | 82 (30.9) | 0.465 | 134 (28.3) | 65 (27.4) | 69 (29.1) | 0.760 | 44 (19.8) | 19 (23.2) | 25 (17.9) | 0.433 | 29 (22.0) | 17 (25.8) | 12 (18.2) | 0.400 |
| Bleeding, n (%) | 64 (9.4) | 30 (7.2) | 34 (12.8) | 0.022 | 46 (9.7) | 18 (7.6) | 28 (11.8) | 0.163 | 52 (23.4) | 17 (20.7) | 35 (25.0) | 0.575 | 31 (23.5) | 16 (24.2) | 15 (22.7) | 1.000 |
| Coinfection, n (%) | 355 (52.3) | 197 (47.6) | 158 (59.6) | 0.003 | 249 (52.5) | 114 (48.1) | 135 (57.0) | 0.066 | 148 (66.7) | 51 (62.2) | 97 (69.3) | 0.350 | 82 (62.1) | 40 (60.6) | 42 (63.6) | 0.858 |
| GCs, n (%) | 265 (39.0) | 0 (0.0) | 265 (100.0) | <0.001 | 237 (50.0) | 0 (0.0) | 237 (100.0) | <0.001 | 140 (63.1) | 0 (0.0) | 140 (100.0) | <0.001 | 66 (50.0) | 0 (0.0) | 66 (100.0) | <0.001 |
| Ribavirin, n(%) | 504 (74.2) | 313 (75.6) | 191 (72.1) | 0.350 | 358 (75.5) | 186 (78.5) | 172 (72.6) | 0.165 | 195 (87.8) | 67 (81.7) | 128 (91.4) | 0.054 | 115 (87.1) | 59 (89.4) | 56 (84.8) | 0.603 |
| immunoglobulin, n(%) | 350 (51.5) | 174 (42.0) | 176 (66.4) | <0.001 | 271 (57.2) | 116 (48.9) | 155 (65.4) | <0.001 | 174 (78.4) | 44 (53.7) | 130 (92.9) | <0.001 | 96 (72.7) | 36 (54.5) | 60 (90.9) | <0.001 |
| RBC (×10^9^/L) | 4.24 (3.87, 4.63) | 4.24 (3.86, 4.61) | 4.25 (3.88, 4.70) | 0.500 | 4.27 (3.88, 4.62) | 4.31 (3.90, 4.61) | 4.24 (3.88, 4.66) | 0.818 | 4.25 (3.95, 4.63) | 4.20 (3.85, 4.62) | 4.27 (4.00, 4.64) | 0.168 | 4.26 (3.92, 4.61) | 4.22 (3.91, 4.69) | 4.30 (3.99, 4.57) | 0.706 |
| HGB (g/L) | 128.00 (117.00, 141.00) | 127.50 (117.00, 141.00) | 128.00 (118.00, 140.00) | 0.725 | 128.00 (117.00, 140.00) | 128.00 (117.00, 141.00) | 127.00 (118.00, 140.00) | 0.588 | 130.00 (117.25, 143.00) | 127.00 (114.25, 143.00) | 131.00 (120.00, 143.00) | 0.164 | 129.50 (118.00, 143.00) | 128.00 (115.25, 143.00) | 130.00 (120.50, 140.75) | 0.677 |
| WBC (×10^9^/L) | 3.16 (1.98, 5.75) | 3.24 (2.08, 5.69) | 3.11 (1.81, 5.76) | 0.167 | 2.97 (1.84, 5.52) | 2.90 (1.85, 5.40) | 3.12 (1.79, 5.76) | 0.979 | 2.94 (1.65, 4.66) | 2.59 (1.58, 4.41) | 3.12 (1.81, 4.97) | 0.172 | 2.55 (1.52, 4.61) | 2.59 (1.55, 4.57) | 2.31 (1.48, 4.66) | 0.933 |
| Neutrophils (%) | 1.91 (1.10, 4.15) | 1.86 (1.11, 4.11) | 2.02 (1.10, 4.18) | 0.801 | 1.90 (1.07, 3.99) | 1.89 (1.03, 3.88) | 1.90 (1.10, 4.13) | 0.601 | 1.98 (1.09, 3.84) | 1.75 (1.05, 3.34) | 2.10 (1.19, 4.07) | 0.151 | 1.79 (1.01, 3.61) | 1.75 (1.09, 3.31) | 1.85 (0.92, 3.82) | 0.743 |
| Lymphocytes (%) | 0.69 (0.48, 1.14) | 0.80 (0.52, 1.24) | 0.61 (0.43, 0.89) | <0.001 | 0.63 (0.44, 0.94) | 0.65 (0.45, 0.98) | 0.61 (0.43, 0.93) | 0.363 | 0.49 (0.33, 0.78) | 0.53 (0.34, 0.78) | 0.48 (0.33, 0.74) | 0.334 | 0.49 (0.34, 0.76) | 0.53 (0.35, 0.80) | 0.46 (0.32, 0.68) | 0.131 |
| Monocytes (%) | 0.17 (0.10, 0.39) | 0.22 (0.11, 0.41) | 0.15 (0.08, 0.30) | <0.001 | 0.15 (0.08, 0.33) | 0.16 (0.09, 0.34) | 0.14 (0.07, 0.30) | 0.280 | 0.08 (0.05, 0.17) | 0.07 (0.05, 0.11) | 0.09 (0.05, 0.17) | 0.359 | 0.08 (0.05, 0.13) | 0.07 (0.05, 0.11) | 0.08 (0.05, 0.14) | 0.705 |
| PLT (×10^9^/L) | 53.00 (37.00, 72.00) | 56.00 (38.00, 75.00) | 49.00 (34.00, 67.00) | 0.001 | 51.50 (36.00, 69.00) | 53.00 (37.00, 70.00) | 50.00 (35.00, 68.00) | 0.371 | 41.50 (30.00, 53.75) | 42.00 (29.00, 54.00) | 41.00 (31.00, 53.00) | 0.720 | 42.00 (31.00, 56.75) | 42.00 (29.25, 57.75) | 42.00 (32.00, 55.50) | 0.936 |
| TBIL (umol/L) | 9.20 (6.80, 12.25) | 9.50 (7.00, 12.60) | 8.60 (6.60, 11.80) | 0.081 | 8.65 (6.60, 11.80) | 9.00 (6.50, 11.80) | 8.60 (6.60, 11.80) | 0.939 | 8.80 (6.40, 12.47) | 8.65 (6.40, 13.28) | 8.80 (6.57, 11.88) | 0.709 | 9.00 (6.50, 12.60) | 8.85 (6.40, 12.83) | 9.20 (7.03, 12.13) | 0.985 |
| DBIL (umol/L) | 4.30 (3.10, 6.10) | 4.30 (3.10, 6.10) | 4.10 (3.10, 6.00) | 0.424 | 4.05 (3.00, 6.00) | 4.10 (3.00, 5.90) | 4.00 (3.10, 6.10) | 0.98 | 4.20 (3.00, 6.30) | 4.25 (3.00, 6.85) | 4.15 (3.05, 5.78) | 0.647 | 4.15 (3.00, 6.00) | 4.30 (3.02, 6.65) | 4.05 (2.80, 5.60) | 0.33 |
| ALT (U/L) | 66.00 (40.95, 105.80) | 64.25 (40.00, 103.81) | 69.00 (41.40, 113.00) | 0.201 | 64.50 (40.82, 105.00) | 67.00 (42.00, 105.60) | 61.80 (40.00, 104.00) | 0.426 | 77.65 (47.73, 142.00) | 81.50 (52.10, 153.75) | 75.25 (46.65, 129.75) | 0.219 | 77.65 (49.57, 153.25) | 77.65 (52.10, 153.75) | 77.00 (46.80, 140.25) | 0.576 |
| AST (U/L) | 138.00 (80.20, 261.65) | 125.85 (70.10, 233.30) | 173.60 (91.00, 311.00) | <0.001 | 144.40 (85.00, 263.68) | 141.00 (84.20, 253.90) | 146.40 (86.40, 274.00) | 0.676 | 238.60 (130.07, 512.65) | 288.50 (142.10, 579.50) | 222.65 (127.73, 459.00) | 0.229 | 230.95 (142.90, 527.92) | 267.00 (142.10, 570.78) | 214.00 (149.75, 506.40) | 0.481 |
| ALP (U/L) | 66.70 (54.00, 87.00) | 65.00 (54.00, 85.75) | 69.00 (56.00, 90.00) | 0.094 | 66.00 (54.00, 85.75) | 64.00 (52.40, 84.00) | 69.00 (54.00, 88.00) | 0.151 | 71.50 (55.02, 93.00) | 79.50 (59.25, 93.75) | 69.00 (53.00, 91.50) | 0.053 | 75.45 (55.08, 91.00) | 80.00 (61.00, 92.75) | 69.00 (52.00, 86.50) | 0.059 |
| GGT (U/L) | 37.00 (22.00, 76.00) | 36.00 (21.00, 78.00) | 38.30 (23.00, 72.00) | 0.585 | 36.00 (22.00, 72.00) | 35.00 (21.00, 77.00) | 37.00 (22.00, 68.00) | 0.85 | 36.00 (23.10, 81.31) | 48.90 (25.15, 92.50) | 34.20 (23.00, 73.25) | 0.078 | 37.65 (23.30, 79.92) | 49.90 (27.18, 95.50) | 33.30 (23.00, 69.00) | 0.069 |
| ALB (U/L) | 33.50 (30.60, 36.65) | 33.35 (30.60, 36.58) | 33.70 (30.60, 36.70) | 0.490 | 33.57 (30.52, 36.58) | 33.20 (30.20, 36.10) | 34.10 (30.90, 37.20) | 0.015 | 32.55 (29.40, 34.90) | 32.40 (29.40, 34.77) | 32.55 (29.48, 35.05) | 0.891 | 32.55 (29.37, 34.52) | 32.40 (29.18, 34.65) | 32.55 (29.42, 34.22) | 0.641 |
| GLOB (g/L) | 25.40 (22.80, 28.30) | 25.60 (22.92, 28.30) | 25.10 (22.30, 28.30) | 0.224 | 25.20 (22.40, 28.30) | 25.30 (22.80, 28.30) | 25.10 (22.20, 28.40) | 0.585 | 26.20 (23.70, 30.17) | 26.00 (23.70, 30.12) | 26.20 (23.78, 30.28) | 0.922 | 25.95 (23.85, 29.68) | 26.00 (23.95, 30.50) | 25.95 (23.75, 28.95) | 0.607 |
| SCr (umol/L) | 68.80 (58.30, 84.15) | 67.00 (56.78, 81.98) | 73.00 (60.60, 90.80) | <0.001 | 70.65 (59.02, 87.15) | 69.20 (58.00, 85.70) | 71.80 (60.00, 87.90) | 0.292 | 81.00 (67.08, 107.28) | 78.75 (64.25, 112.22) | 81.10 (68.22, 101.25) | 0.803 | 81.00 (66.70, 109.10) | 80.95 (67.47, 118.43) | 81.00 (66.10, 95.60) | 0.495 |
| BUN (mmol/L) | 4.83 (3.50, 6.51) | 4.46 (3.38, 6.10) | 5.30 (3.96, 6.99) | <0.001 | 5.01 (3.70, 6.59) | 4.65 (3.40, 6.30) | 5.20 (3.96, 6.90) | 0.004 | 6.26 (4.61, 8.82) | 5.87 (4.26, 8.79) | 6.50 (4.93, 8.89) | 0.299 | 6.15 (4.28, 8.86) | 6.02 (4.21, 9.18) | 6.44 (4.71, 8.64) | 0.670 |
| LDH (U/L) | 620.00 (402.50, 957.50) | 564.00 (367.25, 888.23) | 725.00 (476.00, 1176.00) | <0.001 | 661.00 (433.25, 1021.25) | 694.00 (413.00, 1009.00) | 648.00 (466.00, 1025.00) | 0.710 | 930.00 (596.75, 1490.75) | 922.00 (542.25, 1367.00) | 930.00 (642.00, 1575.00) | 0.312 | 934.50 (627.75, 1465.25) | 918.00 (546.50, 1430.50) | 936.00 (695.75, 1486.25) | 0.294 |
| CK (U/L) | 436.00 (164.50, 1096.50) | 370.50 (143.25, 968.50) | 544.00 (221.00, 1125.29) | <0.001 | 504.00 (192.00, 1105.00) | 529.00 (191.00, 1125.29) | 496.00 (192.00, 1075.00) | 0.903 | 735.50 (345.75, 1293.00) | 701.50 (333.50, 1247.75) | 748.00 (366.75, 1448.75) | 0.564 | 674.50 (359.00, 1318.75) | 674.50 (334.25, 1268.75) | 657.00 (392.50, 1479.00) | 0.654 |
| Na (mmol/L) | 135.30 (132.30, 138.05) | 135.70 (133.00, 138.20) | 134.90 (132.00, 137.70) | 0.013 | 135.00 (132.00, 137.50) | 135.10 (132.80, 137.60) | 134.50 (131.90, 137.50) | 0.265 | 134.50 (131.10, 138.00) | 135.50 (132.00, 138.78) | 134.00 (131.00, 137.60) | 0.100 | 134.50 (131.10, 137.67) | 135.60 (133.27, 138.95) | 133.60 (130.25, 135.95) | 0.002 |
| K (mmol/L) | 3.62 (3.32, 3.92) | 3.60 (3.32, 3.89) | 3.69 (3.31, 4.00) | 0.013 | 3.63 (3.31, 3.92) | 3.59 (3.31, 3.89) | 3.68 (3.31, 3.97) | 0.054 | 3.76 (3.38, 4.10) | 3.68 (3.34, 4.04) | 3.80 (3.47, 4.10) | 0.278 | 3.71 (3.34, 4.00) | 3.62 (3.32, 4.02) | 3.72 (3.42, 3.99) | 0.562 |
| Ca (mmol/L) | 1.96 (1.88, 2.05) | 1.98 (1.89, 2.07) | 1.94 (1.87, 2.02) | 0.001 | 1.96 (1.88, 2.04) | 1.96 (1.88, 2.04) | 1.95 (1.89, 2.02) | 0.823 | 1.91 (1.80, 2.01) | 1.91 (1.80, 2.00) | 1.92 (1.80, 2.01) | 0.868 | 1.90 (1.79, 2.00) | 1.90 (1.80, 2.00) | 1.90 (1.75, 1.98) | 0.111 |
| D-Dimer (mg/L) | 2.55 (1.25, 5.14) | 1.99 (1.09, 5.14) | 3.21 (1.56, 5.21) | <0.001 | 2.94 (1.40, 5.14) | 2.87 (1.28, 5.14) | 3.02 (1.52, 5.14) | 0.184 | 4.72 (2.55, 8.41) | 4.42 (2.58, 7.50) | 4.82 (2.54, 8.54) | 0.752 | 4.69 (2.60, 8.33) | 5.00 (2.76, 8.46) | 4.52 (2.56, 7.90) | 0.654 |
| PT (s) | 12.20 (11.30, 12.90) | 12.20 (11.20, 12.90) | 12.26 (11.40, 13.00) | 0.242 | 12.10 (11.20, 12.90) | 12.10 (11.10, 12.90) | 12.20 (11.30, 12.90) | 0.647 | 12.20 (11.10, 13.28) | 12.50 (11.33, 13.38) | 12.00 (11.00, 12.93) | 0.072 | 12.33 (11.30, 13.30) | 12.23 (11.10, 13.30) | 12.50 (11.40, 13.38) | 0.759 |
| INR | 0.97 (0.91, 1.03) | 0.97 (0.91, 1.03) | 0.97 (0.91, 1.04) | 0.810 | 0.97 (0.91, 1.03) | 0.97 (0.93, 1.04) | 0.97 (0.91, 1.03) | 0.344 | 1.04 (0.97, 1.11) | 1.03 (0.96, 1.10) | 1.05 (0.97, 1.12) | 0.443 | 1.04 (0.97, 1.12) | 1.04 (0.98, 1.12) | 1.05 (0.97, 1.12) | 0.831 |
| APTT (s) | 43.90 (37.40, 51.25) | 41.90 (36.15, 49.68) | 46.60 (39.90, 54.20) | <0.001 | 45.30 (38.40, 52.38) | 45.30 (38.30, 52.60) | 45.80 (39.10, 51.90) | 0.716 | 54.60 (42.25, 63.90) | 57.50 (43.63, 63.90) | 53.40 (41.00, 63.88) | 0.251 | 57.75 (46.18, 65.40) | 56.55 (43.02, 63.23) | 58.20 (49.15, 66.60) | 0.170 |
| FIB (g/L) | 2.51 (2.18, 2.92) | 2.53 (2.20, 2.94) | 2.48 (2.12, 2.87) | 0.061 | 2.48 (2.13, 2.84) | 2.44 (2.13, 2.80) | 2.53 (2.13, 2.89) | 0.247 | 2.20 (1.80, 2.55) | 2.32 (1.90, 2.72) | 2.10 (1.77, 2.48) | 0.013 | 2.20 (1.80, 2.55) | 2.26 (1.90, 2.68) | 2.17 (1.74, 2.54) | 0.169 |
| TT (s) | 23.20 (19.60, 27.90) | 22.30 (18.90, 27.28) | 24.10 (20.50, 29.30) | 0.001 | 23.50 (20.22, 28.90) | 23.50 (20.30, 30.10) | 23.50 (20.10, 27.50) | 0.678 | 27.05 (20.58, 39.79) | 26.95 (21.12, 41.90) | 27.40 (20.48, 39.27) | 0.674 | 28.60 (21.20, 41.45) | 26.15 (21.12, 41.82) | 30.15 (21.68, 40.95) | 0.646 |
| CRP (mg/L) | 9.00 (3.40, 11.00) | 9.00 (3.30, 11.62) | 9.00 (3.89, 9.92) | 0.647 | 9.00 (3.51, 11.00) | 9.00 (3.40, 12.51) | 9.00 (3.89, 9.64) | 0.245 | 9.00 (9.00, 13.83) | 9.00 (5.22, 13.83) | 9.00 (9.00, 13.83) | 0.239 | 9.00 (8.89, 13.05) | 9.00 (9.00, 13.83) | 9.00 (8.85, 11.20) | 0.395 |
| PCT (ng/L) | 0.26 (0.10, 0.95) | 0.22 (0.10, 1.03) | 0.28 (0.11, 0.91) | 0.043 | 0.28 (0.10, 0.90) | 0.31 (0.10, 1.05) | 0.27 (0.10, 0.80) | 0.729 | 0.58 (0.20, 1.05) | 0.66 (0.24, 1.05) | 0.56 (0.19, 1.05) | 0.423 | 0.62 (0.19, 1.05) | 0.87 (0.24, 1.05) | 0.56 (0.16, 1.05) | 0.249 |
| NLR | 2.76 (1.43, 5.79) | 2.41 (1.32, 5.23) | 3.36 (1.88, 7.00) | <0.001 | 2.97 (1.52, 6.83) | 2.75 (1.45, 6.57) | 3.23 (1.75, 7.00) | 0.195 | 3.74 (2.02, 7.24) | 2.98 (1.80, 6.15) | 4.36 (2.17, 8.20) | 0.016 | 3.34 (1.93, 6.64) | 2.98 (1.87, 5.68) | 3.93 (2.17, 7.70) | 0.097 |
| CAR | 0.24 (0.11, 0.34) | 0.24 (0.11, 0.36) | 0.24 (0.12, 0.32) | 0.729 | 0.24 (0.12, 0.33) | 0.25 (0.12, 0.37) | 0.24 (0.11, 0.31) | 0.179 | 0.28 (0.24, 0.46) | 0.27 (0.18, 0.41) | 0.29 (0.24, 0.49) | 0.117 | 0.28 (0.23, 0.40) | 0.28 (0.23, 0.42) | 0.28 (0.23, 0.38) | 0.792 |
| PLR | 73.00 (43.44, 123.04) | 70.37 (42.48, 116.04) | 81.82 (45.90, 125.00) | 0.082 | 79.68 (46.87, 130.97) | 79.17 (47.58, 135.48) | 82.61 (46.15, 127.87) | 0.998 | 82.89 (47.54, 139.36) | 73.40 (49.00, 130.19) | 84.98 (46.49, 139.69) | 0.436 | 85.15 (49.19, 143.43) | 79.07 (45.97, 140.18) | 89.45 (52.46, 143.33) | 0.249 |
| Lg viral load (copies/ml) | 3.26 (2.00, 5.08) | 2.80 (2.00, 4.54) | 3.78 (2.53, 5.51) | <0.001 | 3.65 (2.16, 5.62) | 3.65 (2.03, 5.92) | 3.58 (2.38, 5.38) | 0.937 | 6.54 (4.53, 7.25) | 5.64 (3.46, 7.24) | 6.87 (5.38, 7.25) | <0.001 | 5.94 (3.61, 7.25) | 5.68 (3.54, 7.25) | 6.20 (3.85, 7.25) | 0.555 |

Table S4 The effect of GC regime on HAI in patients with SFTS after propensity score matching.

| Severity of SFTS | Variables | Levels | HAI (%) | p-Value | OR (95%CI) |
| --- | --- | --- | --- | --- | --- |
| All patients (n =72) | Initiation of GC therapy | > 6 days | 42 (23.73) | 0.987 | - |
|  |  | ≤ 6 days | 30 (23.81) |  | 1.004 (0.584, 1.713) |
|  | Duration of GC therapy | > 3 days | 34 (25.95) | 0.434 | 1.236 (0.725, 2.104) |
|  |  | ≤ 3 days | 38 (22.09) |  | - |
|  | Dose of GC therapy | high | 27 (25.47) | 0.608 | 1.154 (0.661, 1.990) |
|  |  | low | 45 (22.84) |  | - |
| Mild patients (n=46) | Initiation of GC therapy | > 6 days | 29 (20.28) | 0.676 | 1.152 (0.598, 2.276) |
|  |  | ≤ 6 days | 17 (18.09) |  | - |
|  | Duration of GC therapy | > 3 days | 16 (16.84) | 0.415 | - |
|  |  | ≤ 3 days | 30 (21.13) |  | 1.323 (0.683, 2.638) |
|  | Dose of GC therapy | high | 16 (20.51) | 0.764 | 1.110 (0.553, 2.164) |
|  |  | low | 30 (18.87) |  | - |
| Severe patients (n=26) | Initiation of GC therapy | > 6 days | 13 (38.24) | 0.843 | - |
|  |  | ≤ 6 days | 13 (40.62) |  | 1.105 (0.410, 2.989) |
|  | Duration of GC therapy | > 3 days | 18 (50.00) | 0.057 | 2.750 (0.995, 8.097) |
|  |  | ≤ 3 days | 8 (26.67) |  | - |
|  | Dose of GC therapy | high | 11 (39.29) | 0.988 | - |
|  |  | low | 15 (39.47) |  | 1.008 (0.371, 2.771) |

GC doses were calculated as dexamethasone equivalence, with > 5 mg/d defined as high dose and ≤ 5 mg/d defined as moderate dose.

Abbreviations: GC, glucocorticoid; HAI, hospital-acquired infection; OR, odds ratio; CI: confidence interval.
